# Role of Cellular Heparan Sulfate Proteoglycans in Infection of Human Adenovirus Serotype 3 and 35

**DOI:** 10.1371/journal.ppat.1000189

**Published:** 2008-10-31

**Authors:** Sebastian Tuve, Hongjie Wang, Jeffrey D. Jacobs, Roma C. Yumul, David F. Smith, André Lieber

**Affiliations:** 1 Division of Medical Genetics, Department of Medicine, University of Washington, Seattle, Washington, United States of America; 2 Department of Biochemistry and Consortium for Functional Glycomics Core H, Emory University School of Medicine, Atlanta, Georgia, United States of America; 3 Department of Pathology, University of Washington, Seattle, Washington, United States of America; Saint Louis University, United States of America

## Abstract

Species B human adenoviruses (Ads) are increasingly associated with outbreaks of acute respiratory disease in U.S. military personnel and civil population. The initial interaction of Ads with cellular attachment receptors on host cells is via Ad fiber knob protein. Our previous studies showed that one species B Ad receptor is the complement receptor CD46 that is used by serotypes 11, 16, 21, 35, and 50 but not by serotypes 3, 7, and 14. In this study, we attempted to identify yet-unknown species B cellular receptors. For this purpose we used recombinant Ad3 and Ad35 fiber knobs in high-throughput receptor screening methods including mass spectrometry analysis and glycan arrays. Surprisingly, we found that the main interacting surface molecules of Ad3 fiber knob are cellular heparan sulfate proteoglycans (HSPGs). We subsequently found that HSPGs acted as low-affinity co-receptors for Ad3 but did not represent the main receptor of this serotype. Our study also revealed a new CD46-independent infection pathway of Ad35. This Ad35 infection mechanism is mediated by cellular HSPGs. The interaction of Ad35 with HSPGs is not via fiber knob, whereas Ad3 interacts with HSPGs via fiber knob. Both Ad3 and Ad35 interacted specifically with the sulfated regions within HSPGs that have also been implicated in binding physiologic ligands. In conclusion, our findings show that Ad3 and Ad35 directly utilize HSPGs as co-receptors for infection. Our data suggest that adenoviruses evolved to simulate the presence of physiologic HSPG ligands in order to increase infection.

## Author Summary

In this study, we attempted to identify binding receptors that are used by the two human adenovirus (Ad) serotypes 3 and 35. Ad3 uses yet-unknown receptors and is one of the most common Ads causing epidemic conjunctivitis, and respiratory and gastrointestinal diseases. Ad35 uses the complement receptor CD46 as an attachment receptor and mainly causes infections of the kidney and urinary tract. We utilized novel high-throughput techniques in combination with the recombinant viral proteins (fiber knobs), which mediate the initial interaction of Ads with host cells. We found that both serotypes interacted with cellular heparan sulfate proteoglycans (HSPGs). In subsequent assays, we show that HSPGs were not major receptors, but acted as low-affinity co-receptors for both Ad3 and Ad35. Ad3 and Ad35 used different viral proteins in order to interact with HSPGs. Both serotypes, however, used the same regions within HSPGs that show high levels of sulfation and are important for binding of extracellular located physiologic ligands. In summary, we show that Ad3 and Ad35 evolved to “highjack” yet another class of cellular surface molecules that are essential for the function of the target host cells and are ubiquitously expressed. This provides new insights into the emerging picture of the infection mechanism of Ad3 and Ad35.

## Introduction

Human adenoviruses (Ads) have been classified into six species (A to F) currently containing 51 serotypes. Most Ad serotypes utilize the coxsackie-adenovirus-receptor, CAR, as a primary attachment receptor [1]. However, this is not the case for species B Ad serotypes [1]. Species B Ads form two genetic clusters, B1 (Ad3, Ad7, Ad16, Ad21, and Ad50) and B2 (Ad11p, Ad14, Ad34, and Ad35) [2]. This classification of species B partially correlates with tissue tropism but does not indicate receptor usage. Recently, we have suggested a new grouping of species B Ads based on their receptor usage [3]. Group 1: (Ad16, 21, 35, 50) nearly exclusively utilize CD46 as a receptor; Group 2: (Ad3, Ad7, 14) share the same non-identified receptor/s which we refer to as receptor X; Group 3: (Ad11p) preferentially interacts with CD46, but also utilizes receptor X if CD46 is blocked [3]. Importantly, our previous study showed that receptor X is identical for Ad3, 7, 11p and 14 [3]. This novel receptor-usage based grouping system is supported by studies from others and us that also found CD46-usage for Ad serotype 11p, 16, 21, 35 and 50 but not for serotype 3 and 7 [4]–[7]. The finding that Ad11p is the only species B Ad family member that evolved to efficiently use both CD46 and receptor X has also been indicated by other previous studies from Gustafsson et al. and Marttila et al. [4],[7]. Marttila et al. confirmed that CD46 blockade on human cells did not affect Ad3 and Ad7 infection, only partially inhibited Ad11p infection and completely abolished infection by serotype 16, 21, 35 and 50 [4]. For Ad14, Marttila et al. suggested that infection of this serotype might partially depend on CD46, however this finding was apparently not significant as indicated by the margin of error in the Ad14 infection assay of this study [4]. Thus, together with the findings of our studies, it appears that Ad16, 21, 35 and 50 nearly exclusively use CD46, Ad11p uses both CD46 and receptor X, while Ad3, 7 and 14 only utilize receptor X as attachment receptors for cellular infection.

Several groups recently attempted to identify receptor X, and various candidates such as CD46, CD80, and/or CD86 were suggested [8]–[11]. However, we and others were so far not able to independently verify that one of these surface molecules represent receptor X. Furthermore studies from others and us (this study included) actually provide contrary evidence that CD46, CD80 and CD86 are not receptor X [3],[4],[6],[7],[12].

Ads cause continuous outbreaks of acute respiratory disease (ARD) in US military training facilities. Studies conducted between 1999 and 2002 revealed that >95% of Ads isolated from recruits were serotype Ad4. Based on this, the US army reinstated an Ad4 vaccination program. The dominance of Ad4 continued through 2005, followed by a simultaneous emergence of diverse species B serotypes at the majority of sites. This included the group 1 serotypes 21 and the group 2 serotypes 3, 7, and 14 [13],[14]. Ad14 outbreaks also occurred in the civil population. During March–June 2007, a total of 140 cases of confirmed Ad14 respiratory illness were identified in clusters of patients in Oregon, Washington and Texas. Thirty eight percent of these patients were hospitalized, including 17% who were admitted to intensive care units (ICUs); 5% of patients died [15]. Furthermore, ARD caused by outbreaks of Ad35 were reported in the past [16],[17].

Species B-derived, replication-deficient vectors (in particular Ad5/3 and Ad5/35 capsid/fiber chimeric vectors) have recently shown promises as vehicles for gene transfer into multiple human cell types including cancer cells and tissue stem cells [18],[19]. In contrast to most human Ads, the infection mechanism and cellular attachment receptor/s of several B species serotypes, in particular Ad3, 7, and 14, have been elusive so far. Considering the emergence of diverse species B Ads as a critical pathogen and the potential practical importance of species B based vectors for gene therapy, we attempted to identify the cellular receptors that are used by species B Ads in addition to CD46. We focused in this study on species B serotypes 3 and 35 that are representative for group 1 and group 2 Ads.

The outer protein capsid of Ads consists of 240 trimeric hexon capsomers, 12 pentameric penton base capsomers and 12 trimeric fibers projecting from the vertices of the icosahedral capsid and ending with a C-terminal fiber knob domain (knob). The knob domain has been identified as a major determinant for the initial cellular attachment of Ads to host cells. We therefore set out to discover yet-unknown Ad3 and Ad35 receptors using the corresponding recombinant fiber knobs.

We identified cellular heparan sulfate proteoglycans (HSPGs) as the main ligand of Ad3 knob but not Ad35 knob. HSPGs were, however, not the main high-affinity receptor for Ad3 (receptor X). Ad3 interacted in a low-affinity manner via fiber knob with HSPGs in order to increase interaction with receptor X (Ad3 co-receptor function of HSPGs). Additionally, we identified a new HSPG-dependent mechanism of Ad35 infection, which was not mediated by the Ad35 fiber knob and was independent of CD46 (Ad35 receptor function of HSPGs). Together, this study shows that both serotypes evolved to utilize HSPGs as co/receptors for infection.

HSPGs typically consist of long polyanionic heparan sulfate (HS) chains (repeating disaccharide units of N-acetylglucoseamine and glucoronic/iduronic acid), which are covalently linked to a protein core (mostly membrane proteins, in particular glypicans, syndecans and CD44v3) [20]. During HSPG biosynthesis successive modification via N-deacetylation-N-sulphatation, epimerization, 2-O-sulphation, 6-O-sulphation and 3-O-sulfation result in a high structural variety in HS-chains. This allows HSPGs to bind to a wide range of proteins (including FGF and TGF family members) [20]–[22]. The classic view of HSPGs is that they serve as co-receptors that bind via their HS chain to various ligands and promote interaction and subsequent signaling via the cognate membrane localized ligand receptors [21]. One example is fibronectin, which binds with different domains to HS-chains of syndecans and to integrins to induce cell spreading and focal adhesion formation [23]. Another example is FGF that requires binding to both, HS-chains and FGF receptor to efficiently induce signaling and endocytosis. HS-chains typically show regions with high, intermediate and low sulfation [24]. In particular, highly sulfated HSPG regions have been shown to participate in the binding of physiologic ligands [20],[22],[25]. HSPGs are also exploited as co/receptors by a wide spectrum of viruses and other parasites. Within the family of human adenoviruses, HSPG interaction has been described for two other serotypes (Ad2 and Ad5) so far [26]. HSPG-Ad5 interaction is via fiber knob and has been proposed to trigger macropinocytosis and subsequent uptake into natural target cells, in particular, lacrimal acini cells [27] and similar observations have been made for the uptake of Ad2 into epithelial cells [28]. Importantly, Ad3 has recently also been shown to utilize macropinocytosis as an uptake mechanism into host cells [29]. These findings, together with the data reported in the present study suggest a general role of HSPGs in Ad infection.

## Results

### Ad3 but not Ad35 fiber knob interacts with cellular HSPGs

**Figure 1:**
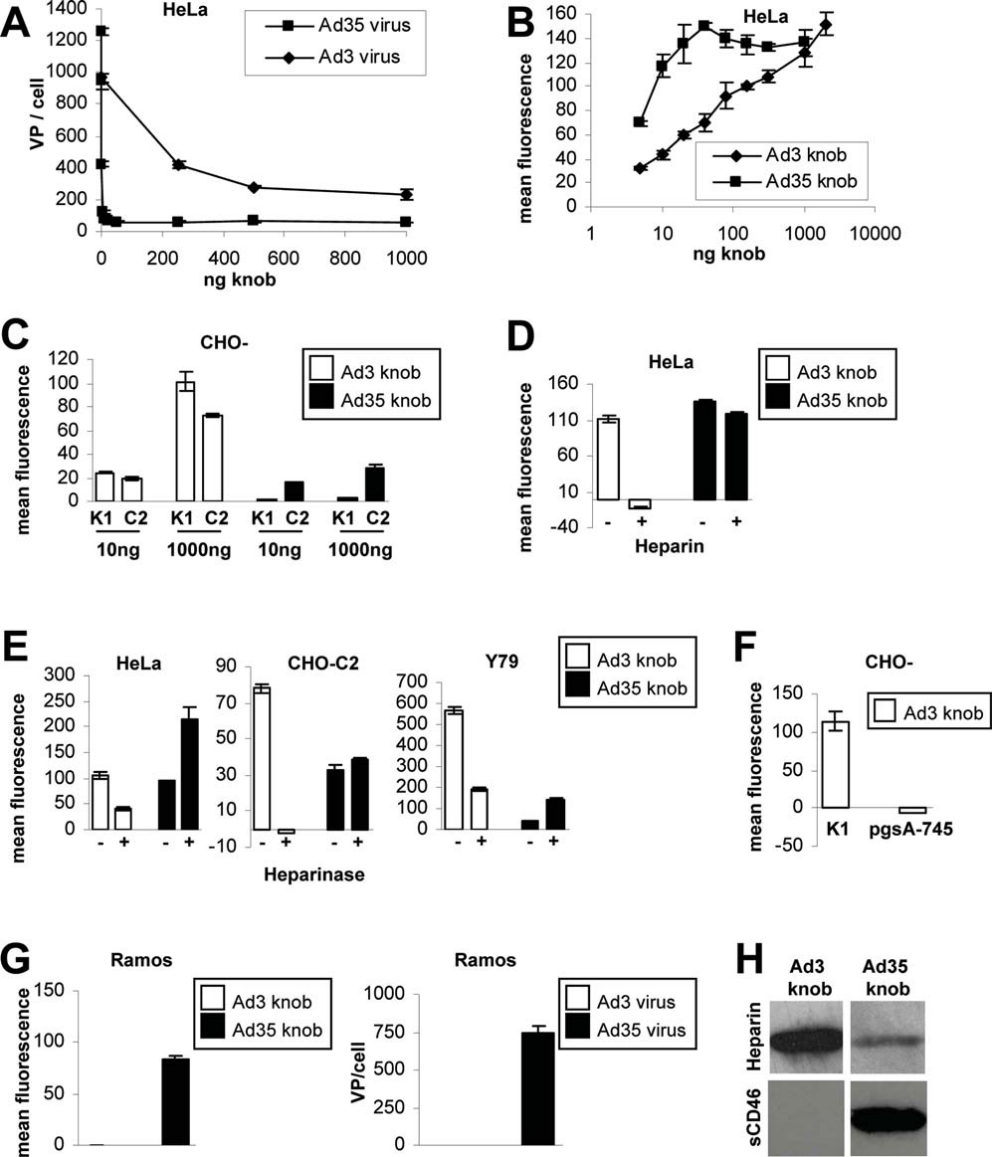
Ad3 but not Ad35 fiber knob interacts with cellular HSPGs. (A) Competition of Ad3 and Ad35 virus particle attachment to HeLa cells using pre-incubation of cells with increasing concentrations of the corresponding knob proteins. (B) Ad3 and Ad35 fiber knob binding to HeLa cells. Note that Ad35 but not Ad3 knob reached saturation of available receptors (for representative flow charts see Figure S2). (C) Ad3 and Ad35 knob binding to CHO-K1 and CHO-C2 cells. (D) Heparin competition of Ad3 and Ad35 knob binding to HeLa cells. (E) Heparinase competition of Ad3 and Ad35 knob binding to HeLa, CHO-C2, and Y79 cells. (F) Ad3 knob binding to CHO-K1 and CHO-pgsA-745 cells. (G) Ad3 and Ad35 knob and virus particle binding to Ramos cells. (H) Ad3 and Ad35 knob binding to Heparin and soluble CD46 assessed via western blot. (A–G) Data points represent the mean and standard deviation of experiments performed in triplicate. All experiments were independently repeated at least once with a similar outcome.

For protein receptor identification we used recombinant trimeric Ad3 and Ad35 knobs for pull down assays using purified HeLa membrane proteins as described before [12]. (HeLa cells express both CD46 and receptor X at high levels [3]). Mass spectrometry analysis of pulled down protein revealed CD46 as an interacting membrane protein for Ad35 knob, which is in agreement with our earlier study [12]. However, for Ad3 knob no valid interacting membrane protein/s could be identified (data not shown). We therefore tested the functionality of purified Ad3 and Ad35 knob via competition for cellular attachment with the corresponding viruses. Pre-incubation of HeLa and 293 cells with the recombinant knobs blocked attachment of the corresponding viruses (Figure 1A and Figure S1). This indicated that the knobs of both viruses are major determinants for attachment of the corresponding viruses. Overall, Ad3 knob showed different blocking properties as compared to Ad35 knob: (i) Ad35 knob reduced binding of Ad35 virus particles by ∼90% (293 cells) and 95% (HeLa cells) at relatively low concentrations (10 ng Ad35 knob/10^5^ cells = 9.4×10^4^ Ad35 knob trimers per cell); and (ii) Ad3 knob reduced binding of Ad3 virus particles only 63% (293 cells) and 76% (HeLa cells) and 50-fold higher concentrations (500 ng Ad3 knob/10^5^ cells = 4.7×10^6^ Ad3 knob trimers per cell) were required for this effect. Next, we incubated HeLa cells with an increasing amount of Ad3 and Ad35 knob and detected the amount of bound knob via flow cytometry. In contrast to Ad35 knob an approximately 50-fold higher concentration of Ad3 knob was necessary to reach a similar amount of knob binding to HeLa cells and no saturation point of Ad3 knob binding was observed, whereas Ad35 knob reached a saturation of binding to HeLa cells at 40 ng knob/10^5^ cells (Figure 1B and Figure S2). In summary, both knobs bound to human cells and competed the binding of the corresponding viruses. However, in contrast to Ad35 knob the Ad3 knob could not be used to identify an interacting membrane protein using mass spectrometry analysis.

Since we did not identify a valid Ad3 knob interacting membrane protein via mass spectrometry analysis we next hypothesized that Ad3 knob might interact with carbohydrates. To test this hypothesis we utilized human and non-human cells. Ad3 knob significantly bound to Chinese hamster ovarian cells (CHO-K1), whereas Ad35 knob only bound to these cells when they were transformed to express human CD46 (CHO-C2; Figure 1C). Removal of sialic acids from the cellular surface of HeLa cells did not reduce Ad3 or Ad35 knob binding, whereas FITC-labeled wheat germ agglutinin (that specifically interacts with sialic acid) showed significantly reduced binding (∼50%, Figure S3). Next we tested whether pre-incubation of knobs with Heparin might abrogate their attachment to cells. Ad3 knob binding was completely blocked, whereas Ad35 knob binding only minimally affected by Heparin (Figure 1D). Heparin is similarly structured to the heparan sulfate (HS) side chains of heparan sulfate proteoglycans (HSPGs), but generally displays higher levels of sulfation as compared to HSPGs [22]. Therefore, we then pretreated HeLa cells with Heparinase I in order to test whether cellular HSPGs might interact with Ad3 knob. Heparinase I reduced HSPG levels on HeLa, CHO-C2 and Y79 cells (72%, 79% and 75% decreased HSPG levels, respectively (data not shown). Importantly, Ad3 knob binding was also reduced upon this treatment to similar extends (HeLa, 61%; CHO-C2, 102%; Y79, 66% decreased Ad3 knob binding, respectively) (Figure 1E). In contrast, for Ad35 knob increased levels of binding were detected on all cell lines upon HSPG removal from the cellular surface. Together these data indicated that Ad3 knob binds to HSPGs on human and hamster cells whereas HSPGs had an inhibitory effect on Ad35 knob attachment to cells.

**Figure 2:**
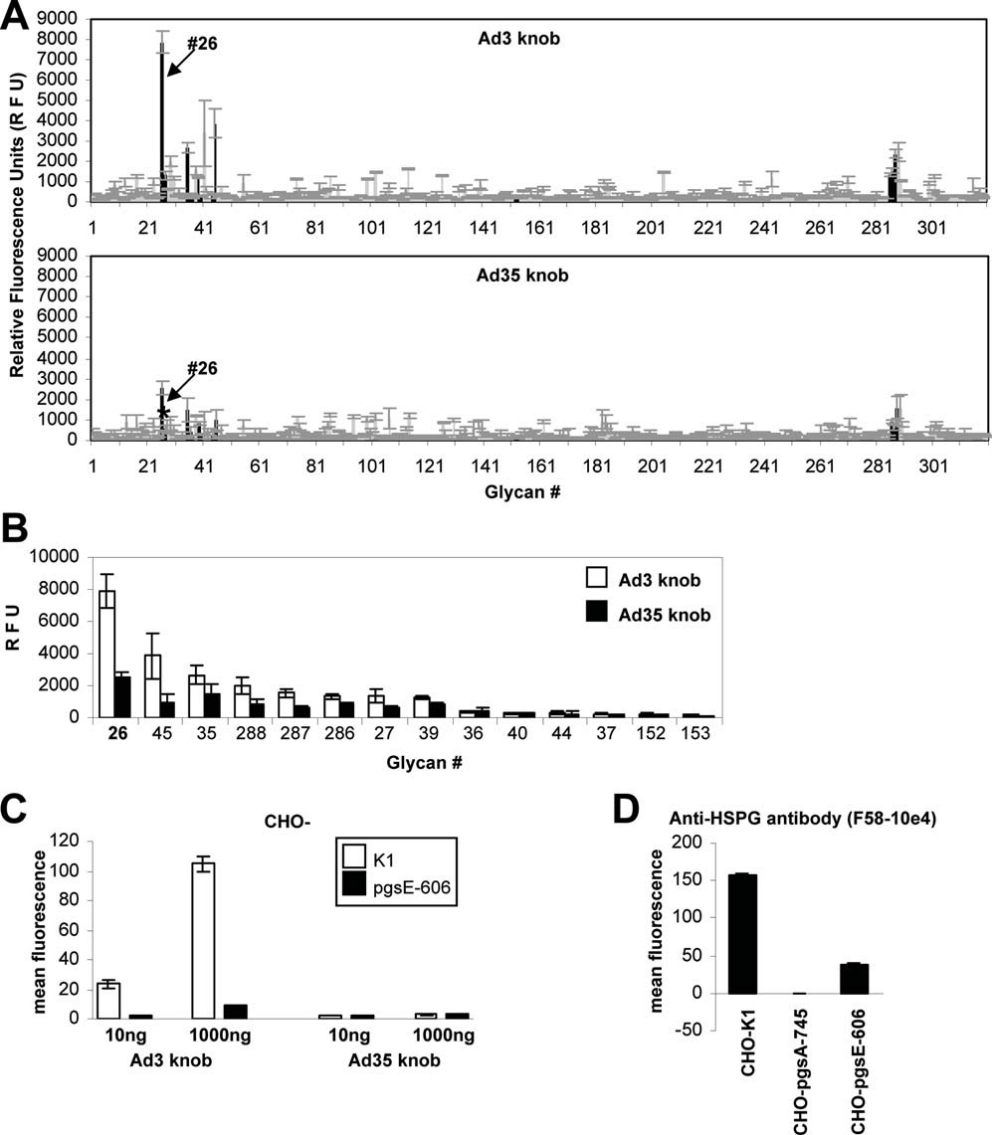
Sulfation of HSPGs is essential for interaction with Ad3 fiber knob. (A) Glycan binding specificity of Ad3 and Ad35 knob on glycan array. The plot shows the average relative fluorescence units (RFU; *y*-axis) for the six addresses of each glycan versus glycan number (*x*-axis) as bars. Standard deviations in the fluorescence for the six addresses are indicated for each glycan. Sulfated glycans are indicated with black bars. Arrows indicate glycan#26. (B) Ad3 and Ad35 knob binding specificity to sulfated glycans on glycan array. For structure of sulfated glycans see Table 1. (C) Ad3 and Ad35 knob binding to CHO-K1 and CHO-pgsE-606 cells. (D) Binding of anti-HSPG antibody to CHO-K1, CHO-pgsA-745, and CHO-pgsE-606 cells. (C,D) Bars represent the mean and standard deviation of experiments performed in triplicate. These experiments were independently repeated once with a similar outcome.

To further investigate the possibility of HSPGs being a receptor for Ad3 knob we utilized CHO cells that are specifically HSPG-negative due to Xylosyltransferase deficiency (CHO-pgsA-745 [30]). These cells did not bind anti-HSPG antibody, whereas native CHO-K1 cells show high levels of anti-HSPG antibody staining. Intriguingly, CHO-pgsA-745 cells did not bind any Ad3 knob at all, which is in stark contrast to native HSPG-positive CHO-K1 cells (Figure 1F). In addition, Ramos cells (which totally lack HSPG expression, Figure S4) do not bind Ad3 knob or Ad3 virus particles (Figure 1G). Interestingly, Ramos cells expressed CD46 and CD86 (Figure S4) and efficiently bound Ad35 knob and virus particles (Figure 1G). Finally, we used 1 µg of each fiber knob and soluble CD46 and Heparin in a highly sensitive western blot assay. Ad35 knob efficiently bound to soluble CD46, whereas Ad3 knob did not show any binding of soluble CD46 at all (Figure 1H). However, Ad3 knob showed efficient binding to Heparin. To our surprise Ad35 also bound to Heparin in the western blot assay, although to a lesser degree as compared to Ad3 knob. However, the biologic relevance of the detected Ad35 knob interaction with Heparin might be questionable since the cell-based assays did not show any receptor function of HSPGs for Ad35 knob, in particular (i) HSPG removal from cells actually increased attachment of Ad35 knob (probably due to better access to the high-affinity ligand CD46 after HSPG removal (Figure 1C)) and (ii) Ad35 knob (in contrast to Ad3 knob) did not efficiently attach to CHO-K1 cells (these cells have high HSPG-levels (Figure 2D) but lack the high-affinity ligand CD46 (Figure 1C)). Overall the western blot assay confirmed our finding that Ad3 knob interacts with Heparin/HSPGs.

Together these data indicated that Ad3 knob binds to HSPGs on cells in a specific and low-affinity manner. In contrast, Ad35 knob interacted with CD46 in a high-affinity manner and HSPGs had an inhibitory effect on Ad35 knob binding to CD46.

### Sulfation of HSPGs is essential for interaction with Ad3 fiber knob

Since Ad3 knob directly interacted with HSPGs, we used a glycan array in order to screen for further carbohydrates that might be used by the Ad3 or Ad35 knobs. The glycan array currently consists of 320 natural and synthesized glycans that are linked to a glass slide. After Ad3 and Ad35 knob incubation on the glass slides and detection of the relative amount of bound knob via primary and secondary AlexaFluor488-labeled antibody, glycan#26 showed the highest level of binding for Ad3 knob (Figure 2A). Ad35 knob also bound to this glycan, although to overall lower levels. Importantly, glycan#26 is the only glycan in the array that has a total of 3 sulfate groups. All other glycans in the array have less or no sulfates (Table 1). Disaccharides that were structured identical, with the only difference being reduced sulfation, bound significantly less Ad3 knob (e.g. glycan#35, 45, 288, 287, 286, etc.; Figure 2B and Table 1). There was a direct correlation of reduced sulfation status (absence of one, two or all sulfate groups) and reduced Ad3 knob binding (Figure 2B and Table 1). Together the array data strongly indicated that the sulfation status of glycans is crucial for Ad3 knob binding. It is important to mention that the array also contained glycans with sialic acid and that these glycans did not show any significant Ad3 or Ad35 knob binding, which argues against a charge-mediated interaction of Ad3 knob with HSPGs (both, sialic acid and HSPGs are negatively charged at neutral pH).

**Table 1:** Structure of sulfated glycans in the glycan array.

Glycan#	Glycan Structure					Spacer	Total Sulfates
26	[3OSO3][6OSO3]	Gal	β1–4	[6OSO3]	GlcNAc	β-Sp0	3
45	[6OSO3]	Gal	β1–4	[6OSO3]	GlcNAc	β-Sp8	2
35	[3OSO3]	Gal	β1–4	[6OSO3]	GlcNAc	β-Sp8	2
288	[6OSO3]	Gal	β1–4	[6OSO3]	GlcNAc	β-Sp0	2
287	[3OSO3][4OSO3]	Gal	β1–4		GlcNAc	β-Sp0	2
286	[3OSO3]	Gal	β1–4	[6OSO3]	GlcNAc	β-Sp0	2
27	[3OSO3][6OSO3]	Gal	β1–4		GlcNAc	β-Sp0	2
39	[4OSO3][6OSO3]	Gal	β1–4		GlcNAc	β-Sp0	2
36	[3OSO3]	Gal	β1–4		GlcNAc	β-Sp0	1
40	[4OSO3]	Gal	β1–4		GlcNAc	β-Sp8	1
44	[6OSO3]	Gal	β1–4		GlcNAc	β-Sp8	1
37	[3OSO3]	Gal	β1–4		GlcNAc	β-Sp8	1
152		Gal	β1–4		GlcNAc	β-Sp0	0
153		Gal	β1–4		GlcNAc	β-Sp8	0

(Ad3 and Ad35 knob binding to these glycans is shown in Figure 2B). Glycan#, glycan number in the array; [3OSO3], 3-O-sulfation; [6OSO3], 6-O-sulfation; Gal, Galactose; GlcNAc, N-acetylglucosamine; β-Sp0, β-CH_2_CH_2_NH_2_ spacer arm connecting glycan to glass slide; β-Sp8, β-CH_2_CH_2_CH_2_NH_2_ spacer arm connecting glycan to glass slide; β1–4, β1–4 glycosidic bond.

To test the possibility that Ad3 knob uses highly sulfated regions within cellular HSPGs, we utilized CHO cells that are Heparan sulfate N-sulfotransferase deficient (CHO-pgsE-606, [31]). These cells express HSPGs that are grossly non-sulfated. Incubation of these cells with a primary mAb against HS that reacts with an HS epitope that is destroyed by N-desulfation [32] showed ∼75% reduced binding as compared to native CHO-K1 cells (Figure 2D). Ad3 knob binding to CHO-pgsE-606 cells was also greatly reduced (∼90%) as compared to CHO-K1 cells (Figure 2C), which confirmed our finding on the glycan array that Ad3 knob specifically interacts with sulfated HSPGs but not with non-sulfated HSPGs. Ad35 knob attached with no quantitative difference and at nearly non-detectable levels to both CHO-K1 and CHO-pgsE-606 cells.

Together these data indicated that Ad3 knob, but not Ad35 knob, binds to highly sulfated regions within cellular HSPGs.

### Effect of HSPG-expression and HSPG-sulfation on Ad3 and Ad35 virus particle attachment to cells

**Figure 3:**
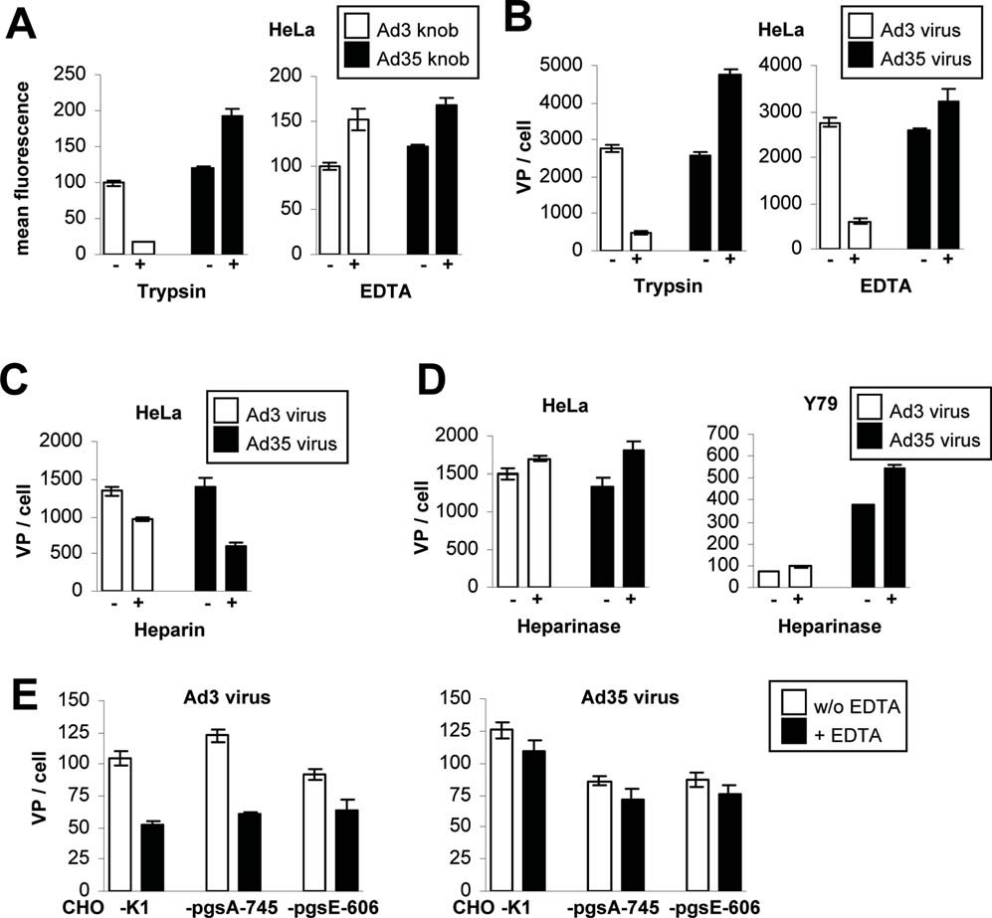
Effect of HSPG-expression and HSPG-sulfation on Ad3 and Ad35 virus particle attachment to cells. (A) Trypsin and EDTA competition of Ad3 and Ad35 knob binding to HeLa cells. (B) Trypsin and EDTA competition of Ad3 and Ad35 virus particle binding to HeLa cells. (C) Heparin competition of Ad3 and Ad35 virus particle binding to HeLa cells. (D) Heparinase competition of Ad3 and Ad35 virus particle binding to HeLa and Y79 cells. (E) EDTA competition of Ad3 virus particle binding to CHO-K1, CHO-pgsA-745, and CHO-pgsE-606 cells. Bars represent the mean and standard deviation of experiments performed in triplicate. All experiments were independently repeated at least once with a similar outcome.

Since we identified sulfated HSPGs as a major cellular receptor for Ad3 knob, we next tested whether this interaction would also be required for the attachment of the corresponding Ad3 virus particles. Our earlier studies showed that Ad3 virus particles interact with receptor X in a trypsin and cation-dependent (EDTA-sensitive) manner [3]. We therefore hypothesized that binding of the Ad3 knob to human cells would also be ablated by these agents. Indeed, trypsin pretreatment of HeLa cells reduced both HSPG levels (data not shown) and binding of Ad3 knob and Ad3 viral particles by ∼80% (Figure 3A and 3B). However EDTA-pretreatment of cells had no inhibitory effect on Ad3 knob binding (Figure 3A). This is in contrast to binding of Ad3 virus particles, which was reduced 78% by the same EDTA concentration (Figure 3B; similar observation on A549 cells, data not shown). We next tested whether Heparin pre-incubation of Ad3 virus particles might decrease virus attachment to HeLa cells. However, the same concentration that completely ablated Ad3 knob binding (Figure 1D) reduced Ad3 virus particle binding only 27% (Figure 3C). Ad35 virus particle binding was even reduced to a greater extent (57%) via pre-incubation with the same Heparin concentration (Figure 3C). Next, we tested whether Heparinase I pre-treatment of cells would have an impact on Ad3 virus particle binding. In contrast to the Ad3 knob (Figure 1E), Heparinase I pretreatment did not reduce Ad3 virus particle attachment to HeLa or Y79 cells, but slightly increased the number of Ad3 virus particles attached per cell (HeLa: 13% increase, *P* = 0.072; Y79: 27% increase, *P* = 0.017; Figure 3D). Ad35 virus particle attachment showed an even higher increase upon Heparinase I treatment of cells (HeLa: 34% increase, *P* = 0.057; Y79: 45% increase, *P* = 0.0054; Figure 3D). Altogether, this indicated that HSPGs have an inhibitory effect on Ad3 and Ad35 virus particle attachment to cells. To further study the role of HSPGs in Ad3 and Ad35 virus particle attachment we next used CHO-pgsA-745 and CHO-pgsE-606 cells that are grossly deficient for HSPG expression and HSPG sulfation, respectively. Overall, Ad3 and Ad35 virus both attached at comparatively low levels to CHO cells (e.g. ∼15-fold lower as compared to HeLa cells) (Figure 3E). Ad3 attached to CHO cells in an EDTA-sensitive manner, which was not observed for Ad35 (Figure 3E) indicating that both serotypes utilize different mechanisms for attachment. For Ad3 the highest binding levels were observed for CHO-pgsA-745 cells followed by CHO-K1 and CHO-pgsE-606 cells (CHO-pgsE-606 versus CHO-pgsA-745: 34% increase, *P* = 0.025). For Ad35 virus the highest attachment levels were observed for CHO-K1 cells as compared to CHO-pgsA-745 and CHO-pgsE-606 cells (CHO-pgsA-745 versus CHO-K1: 46% increase, *P* = 0.012; CHO-pgsE-606 versus CHO-K1: 44% increase, *P* = 0.019).

In summary, these data show that cellular HSPGs were not essential for Ad3 or Ad35 attachment to cells.

### Effect of HSPG-expression and HSPG-sulfation on Ad3 and Ad35 infection of CHO cells

After studying the role of HSPGs in Ad3 and Ad35 knob and virus particle attachment we next investigated the role of HSPGs in infection by these serotypes. We did not observe any difference in Ad3 and Ad35 induced CPE formation between Heparinase I and the mock pre-treated HeLa or A549 cells as determined via crystal violet and MTT assay (data not shown). However, this result was not surprising since HSPGs have a relatively short half-life on the cellular surface (3–8 h), and are either (i) shed by the action of proteases or specific phospholipases for GPI-linked HSPGs or (ii) taken up by endocytosis and recycle back to the surface or can be degraded in the lysosomes, which altogether results in a continuous renewal of cell surface located HSPGs (a process that is facilitated in infection assays at 37°C, but inhibited in attachment assays at 4°C) [22],[25]. Altogether, we conclude that in contrast to attachment assays, in infection assays Heparinase I pre-treatment is not a sufficient model. Overall these data show that partial removal of HSPGs via Heparinase I had no effect on Ad3 and Ad35 infection.

**Figure 4:**
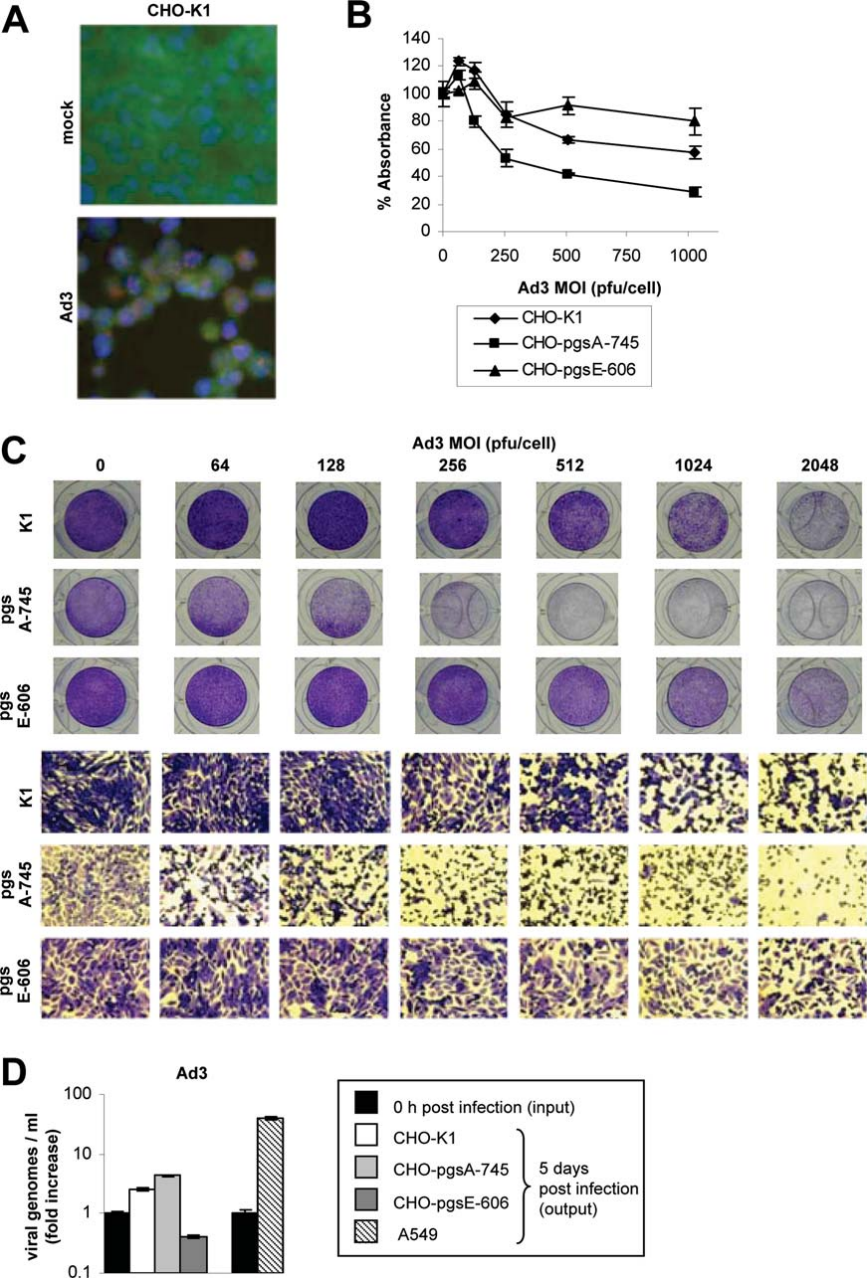
Effect of HSPG-expression and HSPG-sulfation on Ad3 infection of CHO cells. (A) Hexon staining. CHO-K1 cells were infected with Ad3 (MOI 512 pfu/cell). Three days post-infection, cells were fixed and stained for adenovirus hexon protein (red), E-cadherin as a cell surface marker (green), and nuclei (DAPI, blue). Magnification 40×. (B) MTT assay. CHO-K1, CHO-pgsA-745, and CHO-pgsE-606 cells were infected with various MOIs of Ad3 (0, 64, 128, 256, 512, 1024 pfu/cell). Seven days post-infection, mitochondrial activity of cells was determined via MTT assay. Data points represent the mean and standard deviation of experiments performed in triplicate. (C) CPE assay. CHO-K1, CHO-pgsA-745, and CHO-pgsE-606 cells were infected with Ad3 in a range of 0–2048 pfu/cell and monitored for CPE as described in Materials and Methods. Five days post-infection, cells were fixed and stained with crystal violet. Representative pictures are shown. Upper row: Photographs of crystal violet stained wells. Lower row: Photographs (Magnification 40×) of crystal violet stained wells. Presence of CPE is indicated with black borderlines. (D) Ad3 viral replication assay. Fold increase of Ad3 viral genomes 5 days post-infection of CHO-K1, CHO-pgsA-745, CHO-pgsE-606, and A549 cells is shown. Bars represent the mean and standard deviation of experiments performed in duplicate. (B–D) These experiments were independently repeated twice with similar outcomes.

**Figure 5:**
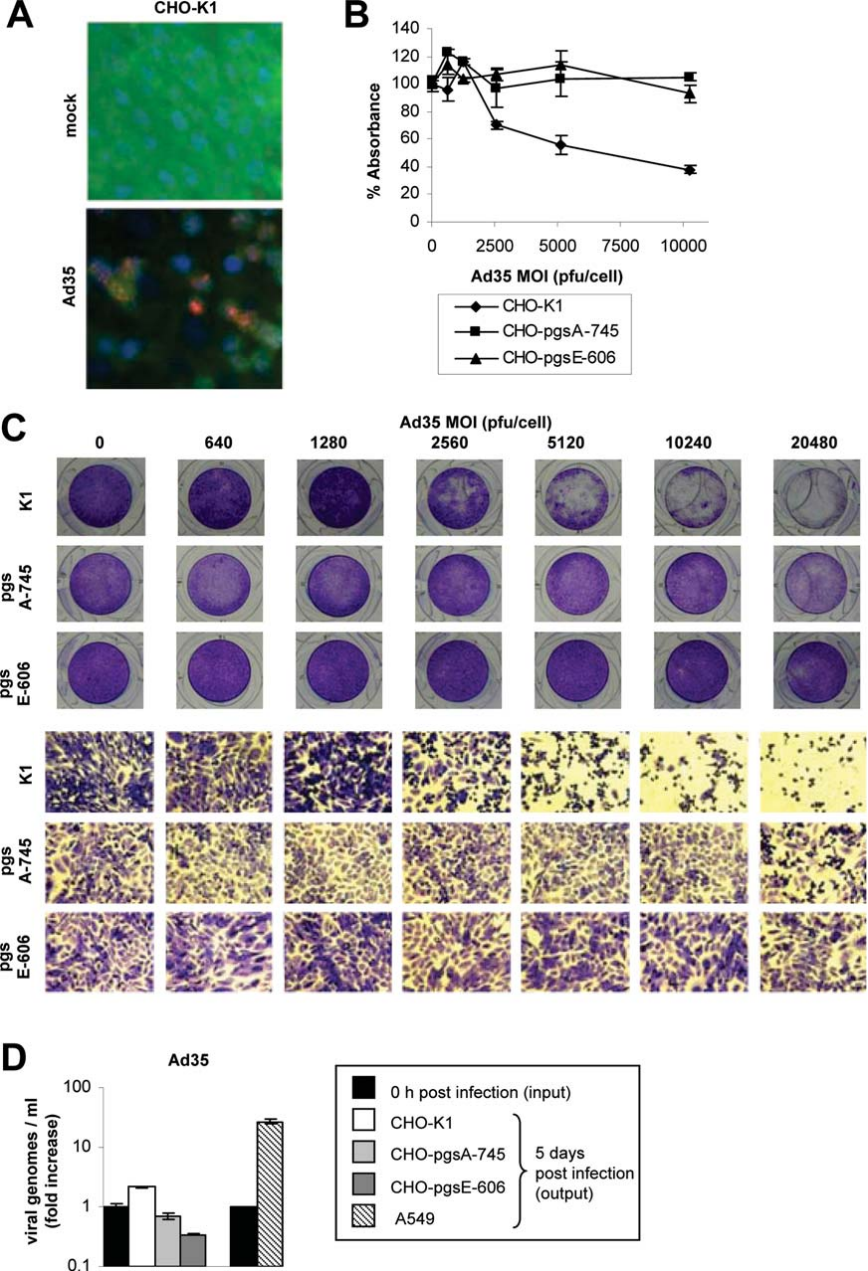
Effect of HSPG-expression and HSPG-sulfation on Ad35 infection of CHO cells. (A) Hexon staining. CHO-K1 cells were infected with Ad35 (MOI 2560 pfu/cell). Three days post-infection, cells were fixed and stained for adenovirus hexon protein (red), E-cadherin as a cell surface marker (green), and nuclei (DAPI, blue). Magnification 40×. (B) MTT assay. CHO-K1, CHO-pgsA-745, and CHO-pgsE-606 cells were infected with an increasing MOI of Ad35 (0, 640, 1280, 2560, 5120, 10240 pfu/cell). Seven days post-infection, mitochondrial activity of cells was determined via MTT assay. Data points represent the mean and standard deviation of experiments performed in triplicate. (C) CPE assay. CHO-K1, CHO-pgsA-745, and CHO-pgsE-606 cells were infected with Ad35 in a range of 0–20480 pfu/cell and monitored for CPE as described in Materials and Methods. Five days post-infection, cells were fixed and stained with crystal violet. Representative pictures are shown. Upper row: Photographs of crystal violet stained wells. Lower row: Photographs (Magnification 40×) of crystal violet stained wells. Presence of CPE is indicated with black borderlines. (D) Ad35 viral replication assay. Fold increase of Ad35 viral genomes 5 days post-infection of CHO-K1, CHO-pgsA-745, CHO-pgsE-606, and A549 cells is shown. Bars represent the mean and standard deviation of experiments performed in duplicate. (B–D) These experiments were independently repeated twice with similar outcomes.

Since Heparinase I pretreatment did not affect adenovirus infection, we next employed native CHO-K1 cells (HSPG expressing), Xylosyltransferase deficient CHO cells (pgsA-745, HSPG-expression deficient) and Heparan sulfate N-sulfotransferase deficient CHO cells (pgsE-606, HSPG-sulfation deficient). A general advantage of these CHO mutants is that they are grossly deficient in HSPG expression (pgsA-745) or HSPG sulfation (pgsE-606) due to enzymatic defects (as compared to their native counterpart CHO-K1) and therefore represent a clear-cut model for investigating the effect of HSPG deficiency. A general disadvantage is that these cells do not express the primary attachment receptor of Ad35 (CD46) and only low levels of receptor X. Low-level expression of receptor X was indicated by Ad3 virus attached to CHO cells, which is less efficient (as compared to human HeLa cells) but also EDTA-sensitive (Figure 3B and 3E). In addition, we observed for Ad serotype 3 and 35 that relatively high MOIs were required to induce CPE in CHO-K1 cells, which is not surprising since human adenoviruses replicate generally less efficient in non-human cells. CPE formation correlated with nuclear Ad hexon staining in Ad3 and Ad35 infected CHO-K1 cells (determined 3 days post-infection). Ad3 induced CPE formation and positive nuclear hexon staining at a minimum MOI of 512 plaque-forming-units (pfu)/cell in CHO cells (Figure 4A and 4C). However, a ∼5-fold higher MOI (2560 pfu/cell) of Ad35 was required for the same effect in CHO-K1 cells (Figure 5A and 5C). This result indicated that CHO-K1 cells were more susceptible towards Ad3 infection. We next investigated the effect of HSPG-expression deficiency in infection by both serotypes using CHO-pgsA-745 cells. As readout for Ad infection we used CPE formation (defined as described in Material and Methods; Figures 4C and 5C). As readout for Ad-induced cell death we used a MTT assay (mitochondrial activity of cells; Figures 4B and 5B). When compared to native CHO-K1 cells, CHO-pgsA-745 cells were markedly more susceptible towards Ad3 infection. In contrast, CHO-pgsA-745 cells were more resistant towards Ad35 infection, when compared to native CHO-K1 cells. Next we tested the effect of HSPG-sulfation deficiency in infection by both serotypes in CHO-pgsE-606 cells. For Ad35 a similar inhibitory effect on infection was observed as seen in HSPG-expression deficiency. Interestingly, for Ad3 an opposite effect of HSPG-sulfation deficiency was observed as compared to HSPG-expression deficiency. In particular, CHO-pgsE-606 cells were more resistant towards Ad3 infection as compared to CHO-K1 cells.

We next tested whether the differences in CPE formation were due to different efficacy of viral uptake or viral replication in these CHO cell lines. First we investigated Ad3 and Ad35 viral particle internalization (Figure S5). Ad3 showed highest internalization levels in CHO-pgsA-745 cells and lowest internalization levels in CHO- pgsE-606 cells. Ad35 showed highest internalization levels in CHO-K1 cells and lower levels of internalization in both CHO-pgsA-745 and CHO-pgsE-606 cells (Figure S5). These data correlates with the CPE (Figures 4C and 5C), MTT (Figures 4B and 5B) and viral attachment data (Figure 3E) and further supports the finding that the susceptibility of CHO cells towards Ad3 and Ad35 infection is directly influenced by the HSPG-expression and -sulfation status. We next analyzed viral replication of Ad3 and Ad35 in CHO cells. In contrast to human A549 cells, CHO cells did not support production of progeny viruses as indicated by decreased numbers of Ad3 and Ad35 plaque forming units 5 days after infection compared to input pfu (Figure S6). However, we found that CHO cells supported replication of Ad3 and Ad35 viral genomes, although viral genome amplification was at least one order of magnitude less efficient as compared to A549 cells (Figures 4D and 5D). Overall, levels of genomic replication correlated with virus attachment and internalization efficacy (Figures 3E and S5) and virus-induced CPE (Figures 4C and 5C) and cell death (Figures 4B and 5B) in CHO cells: Specifically, for Ad3 the highest levels of genomic replication were observed in CHO-pgsA-745 cells (440% increase, *P* = 0.0017), whereas lower replication levels were detected in CHO-K1 cells (153% increase, *P* = 0.0082) and no increase of viral genomes was observed in CHO-pgsE-606 cells (59% decrease, *P* = 0.0037). For Ad35 only CHO-K1 cells showed viral genome amplification (115% increase, *P* = 0.0091), whereas CHO-pgsA-745 cell (31% decrease, *P* = 0.073) and CHO-pgsE-606 cell (67% decrease, *P* = 0.0018) infection did not result in increased Ad35 genome levels. Together, from these data we conclude that the HSPG status of CHO cells influences their susceptibility to Ad3 and Ad35 attachment and internalization, which downstream causes quantitative differences in viral DNA replication, CPE formation and virus induced cell death for both serotypes.

In summary, we show for Ad3 that HSPG expression deficiency increased and lack of HSPG sulfation decreased infection by this serotype. For Ad35 the data show that lack of HSPG expression and lack of HSPG sulfation both inhibited infection.

## Discussion

The aim of this study was to identify novel cellular receptors that are used by species B Ads. For this purpose we employed recombinant Ad3 and Ad35 knob. Screening assays (affinity capture/mass spectrometry and glycan array) indicated that (i) CD46 is a ligand of the Ad35 but not the Ad3 knob and (ii) cellular heparan sulfate proteoglycans (HSPGs) are ligands of the Ad3 but not the Ad35 knob. We subsequently confirmed that the Ad3 but not the Ad35 knob interacted with HSPGs on cells in a cation-independent, sulfation-dependent and low-affinity manner. In contrast to the knob, Ad3 virus particles mainly attached to cells in a cation-dependent, HSPG-independent and high-affinity manner. Therefore our data clearly indicated that HSPGs were not identical to the main Ad3 receptor X. An important conclusion from our data is therefore that the Ad3 knob protein apparently lacks a high-affinity receptor on cells and does not independently interact with the major Ad3 primary attachment receptor. These findings are surprising, since they are in contrast to other adenovirus serotypes, such as Ad2, 5, and 35, for which the knob-interacting proteins have been found by us and others to be identical with the primary attachment receptors of the corresponding viruses [12], [33]–[35]. Since our data indicates that Ad3 knob is not independently responsible for interaction with the main receptor of Ad3 virus, we currently attempt to identify this/these receptor/s X using whole Ad3 viral particles for pull-down assays and subsequent mass spectrometry analysis. We predict that viral particles are more likely to reveal the full spectrum of Ad3 interacting cell surface molecules, as compared to recombinant Ad3 knob.

**Figure 6:**
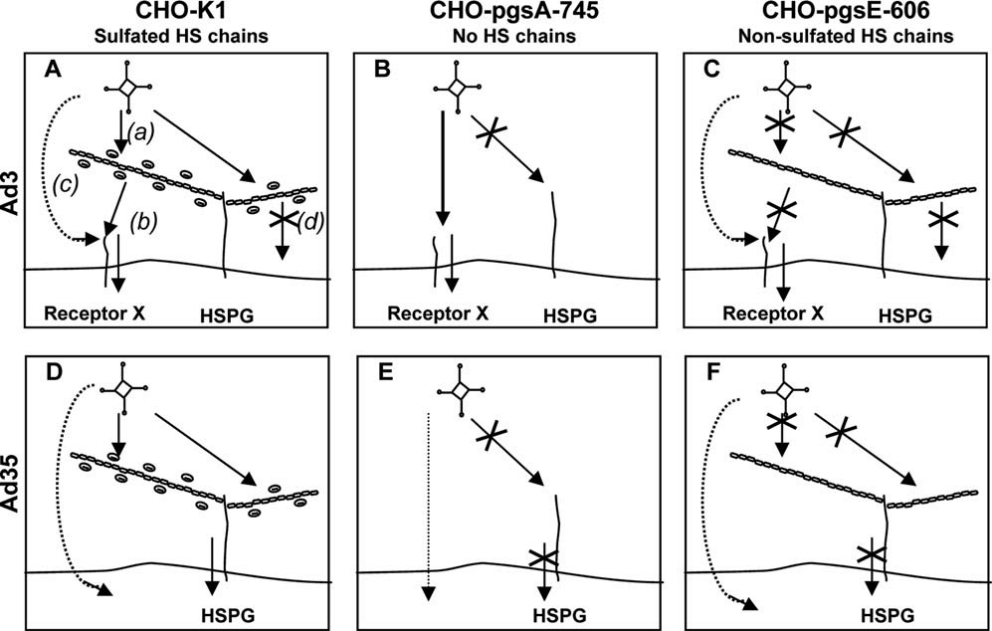
Model of HSPG-function in Ad3 and Ad35 infection of CHO cells. Ad3 and Ad35 infection of CHO cells expressing (i) sulfated HSPGs (CHO-K1), (ii) no HSPGs (CHO-pgsA-745), or (iii) non-sulfated HSPGs (CHO-pgsE-606). (A) Ad3 interacts via fiber knob with sulfated HSPGs (a), which facilitates infection via receptor X (co-receptor function of HSPGs) (b). Ad3 also directly interacts with receptor X (independent of HSPGs) (c). HSPGs that do not co-localize with receptor X also bind Ad3, which does not increase Ad3 infection (barrier function of sulfated HSPGs due to Ad3 binding) (d). (B) Absence of HSPG expression overall increases Ad3 infection. Ad3 directly interacts with receptor X. (C) Ad3 knob does not interact with non-sulfated HSPGs. This blocks Ad3 interaction with HSPGs. Non-sulfated HSPGs do not act as an Ad3 co-receptor. Non-sulfated HSPGs inhibit access of Ad3 to receptor X (physical barrier function of non-sulfated HSPGs). Consequently, Ad3 infection is decreased in CHO-pgsE-606 cells. (D) Ad35 utilizes sulfated HSPGs as alternative low-affinity receptors in the absence of the high-affinity receptor CD46. This mediates infection of CD46-negative CHO cells (receptor function of HSPGs). Absence of HSPG expression (E) and absence of HSPG sulfation (F) strongly decreases Ad35 infection (loss of HSPG receptor function).

Overall, for Ad3 our study provides strong evidence that sulfated HSPGs act as co-receptors for this serotype: (i) High-throughput screening on a glycan array revealed sulfated glycans as the only significant Ad3 knob ligands; (ii) Removal of HSPGs (via Heparinase I pre-treatment of cells) inhibited Ad3 knob attachment to human cells; (iii) HSPG-expression deficiency (CHO-pgsA-745) ablated Ad3 knob attachment; (iv) HSPG-sulfation deficiency (CHO-pgsE-606) ablated Ad3 knob attachment; (v) HSPG-sulfation deficiency (CHO-pgsE-606) inhibited Ad3 virus attachment and infection as compared to native CHO-K1 cells; (vi) Pre-incubation of human cells with Ad3 knob reduced attachment of Ad3 virus (most likely because of competitive inhibition for sulfated binding sites on cellular HSPGs); (vii) The only human cell line (Ramos) that did not express HSPGs was the only human cell line that did not bind any Ad3 knob (all other human cell lines expressed HSPGs and bound Ad3 knob); (viii) Pre-incubation of Ad3 knob with Heparin ablated binding of Ad3 knob to cells; (ix) Pre-incubation of Ad3 virus with Heparin partially inhibited Ad3 virus attachment to cells; and finally (x) In a western blot assay high levels of Heparin (but no soluble CD46) were bound by Ad3 knob. Together these data indicated that Ad3 virus interacts via fiber knob with sulfated HSPGs in order to increase cellular attachment and infection (Ad3 co-receptor function; summarized in Figure 6). Although HSPGs apparently acted as Ad3 co-receptors, part of our data indicated that HSPGs also functioned as a barrier for Ad3 attachment and infection (Figure 6): (i) Removal of HSPGs (via Heparinase I pre-treatment of cells) did not decrease but slightly increased Ad3 virus particle attachment to human cells, and (ii) HSPG expression deficiency (CHO-pgsA-745) markedly increased Ad3 infection as compared to native CHO-K1 cells. In summary, we conclude that our data points towards a dual role of HSPGs in Ad3 infection. We propose that Ad3 evolved to interact with HSPGs via fiber knob (in a sulfation-dependent and low-affinity manner) in order to partially overcome the barrier function of these abundantly expressed cell surface molecules and enhance access to the main receptor/s X (Figure 6).

Several candidate attachment receptors for Ad3 have been recently suggested, including CD46 [8],[11], CD80 and CD86 [9],[10]. However, the data of this study and other previous studies strongly argue against these molecules being identical with the main Ad3 receptor X. Some of these data include: (i) Ramos cells expressed CD46 and CD86 and bound Ad35 knob and virus particles but did not bind Ad3 knob or virus particles at all (this study); (ii) CHO cells did not express CD46, CD80 or CD86 but did bind Ad3 knob and virus particles (this study); (iii) Ad35 knob, but not Ad3 knob, bound to soluble and membrane localized CD46 (this study); (iv) Ad3 virus particles efficiently attached to and infected multiple human cancer cells that did not express CD80 and CD86 and received CD46 blockade [3],[4]; (v) CD46 siRNA reduced Ad35, but not Ad3 attachment to cells [3]; (vi) Soluble CD46 inhibited Ad35 but not Ad3 virus particle attachment to cells [3],[4]. (vii) CD80 and CD86 are co-stimulatory ligands for CD28-mediated T cell activation and are expressed in immune cells (in particular professional antigen-presenting cells upon activation) [36] or certain leukemia cells (e.g. Ramos and K562 cells) but not by epithelial cells that are the natural target of Ad3 infection (this study).

There are, however, several possibilities that could reconcile the findings from other groups that CD46, CD80 and CD86 are utilized as attachment receptors by Ad3 with the contrary data from us and others: (i) One possibility would be that Ad3 indeed interacts with CD46, CD86 and/or CD80 but only with a very low affinity, so that only when very high ectopic receptor expression levels are used in re-expression models (like BHK-CD46, CHO-CD86, CHO-CD80 cells) a measurable increase of Ad3 interaction with the cell occurs. Indeed, in the studies on CD46 by Fleischli et al. and on CD80/86 by Short et al., very high (and arguable non-physiologic) expression levels of these molecules were used on CHO/BHK cells [8],[9]. Notably, while Short et al., reported significant CD80 and CD86 expression on HeLa cells, we were unable to detect these molecules on HeLa cells using flow cytometry. Furthermore, in the study by Fleischli et al. it was reported that a 100-fold higher concentration of soluble CD46 was required to achieve detectable interaction of Ad3 and Ad7 viral particles with this molecule (when compared to Ad11 viral particles) [8]. This indicated that although Ad3 might interact with CD46, the affinity of this interaction might be several orders of magnitude lower as compared to that of Ad11 to CD46. Importantly, we previously found that Ad3 viral particles have a similar (and not a several logs reduced) affinity as compared to Ad11 and Ad35 viral particles to human cells (Ad3 VP K_a_: 3.6e9 M^−1^; Ad11 VP K_a_: 4.3e9 M^−1^; Ad35 VP K_a_: 6.5e9 M^−1^) [3]. In recent studies from us and others the interactions of purified Ad11 and Ad35 knobs with CD46 were found to be of high affinity (Ad11 knob K_D_: 2 nM; Ad35 knob K_D_: 15.5 nM) [35],[37]. We also tried to determine the Ad3 knob affinity to CD46 in the same study [35] and found that it was (if existent) below the sensitivity of the SPR assay (data not shown). The apparently low (if existent) affinity of Ad3 to CD46 together with the general absence of CD80 and CD86 expression on human epithelial cells therefore provide further evidence that a yet-unknown attachment receptor X, and not CD46, CD80 and/or CD86, mainly mediates cellular attachment and infection of Ad3. (ii) A second possibility would be that forced over-expression of CD46, CD80 or CD86 on non-human cells could indirectly increase HSPG and/or receptor X levels (e.g. due to formation of stable complexes or, in case of HS-chains, direct linking); and finally (iii) a third possibility could be that CD46, CD80 and/or CD86 might not be independent attachment receptors but co-receptors for Ad3.

For Ad35 we identified a novel CD46-independent infection mechanism, which is dependent on sulfated HSPGs (Ad35 receptor function of HSPGs; summarized in Figure 6). The following findings for Ad35 supported this conclusion: (i) Pre-incubation of Ad35 virus particles with Heparin markedly reduced Ad35 virus particle attachment to cells; (ii) HSPG-expression deficiency (CHO-pgsA-745) reduced attachment and infection of Ad35 as compared to native CHO-K1 cells; and (iii) Lack of HSPG-sulfation (CHO-pgsE-606) reduced attachment and infection of Ad35 as compared to native CHO-K1 cells. Intriguingly, recombinant Ad35 knob exclusively interacted with CD46 and not with cellular HSPGs, which indicated that the observed Ad35-HSPG interaction is not mediated by the Ad35 fiber knob but via other viral proteins. This conclusion is supported by the following findings for the Ad35 knob: (i) Pre-incubation of Ad35 fiber knob with Heparin reduced only minimally Ad35 knob attachment to cells; (ii) Ad35 knob did not bind to CD46-negative cells that express HSPGs; (iii) Ad35 knob showed only low binding to sulfated glycans in a glycan array; and (iv) Ad35 knob bound strongly to soluble CD46 but only minimally to Heparin in a western blot assay. Altogether, the cell-based assays clearly showed the absence of an Ad35 knob interaction with cellular HSPGs. Our results also showed that, similar to Ad3, HSPGs can act as a physical barrier for Ad35 attachment to cells, in particular pre-incubation of HeLa cells with Heparinase I strongly increased Ad35 knob and Ad35 virus particle attachment. In contrast, on CD46-negative CHO cells sulfated HSPGs acted as alternative receptors (Figure 6). In summary our data indicated that HSPG-dependent (and CD46-independent) Ad35 infection has in general a lower efficacy. In our experiments, the CD46-Ad35 interaction was the dominant mechanism as compared to HSPG-Ad35 interaction (when both CD46 and HSPGs were expressed on the cell). The HSPG-Ad35 interaction therefore had apparently a lower affinity as compared to the CD46-Ad35 interaction. Ramos cells (which expressed CD46, but not HSPGs) bound very efficiently Ad35 knob and viral particles, which showed that HSPGs are not required for Ad35 attachment to CD46 expressing cells. In summary, the data support a dual role of HSPGs in Ad35 infection: They act as alternative low-affinity receptors for CD46-independent infection (in the absence of CD46 expression; summarized in Figure 6) but they also represent a physical barrier between Ad35 and CD46 (in the presence of CD46 expression). Since sulfated HSPGs can act as Ad35 receptors, the barrier function of HSPGs towards CD46 is unlikely to be due to electrostatic repulsion of the Ad35 capsid and we therefore speculate that HSPGs are more likely to physically block access of Ad35 to CD46.

Because CD46 is expressed on all nucleated cells in humans, the question about the relevance of Ad35 binding studies on cells that lack CD46 arises. Considering our conclusion that HSPGs are a barrier to Ad35-CD46 interaction as well as our recent finding that, in primary polarized epithelial cells, CD46 is trapped in tight junctions (Robert Strauss, et al., in preparation), one could speculate that CD46 is not accessible on epithelial tissue *in vivo*. This scenario is not new for adenoviruses. On lung epithelial tissue, CAR, the receptor for most adenoviruses including Ad2 and Ad5, is expressed only on the basolateral surface and access to CAR is blocked by the glycocalix [38],[39]. Interestingly, Ad2 and Ad5 also interact with HSPG with low affinity [26]. We therefore hypothesize that adenoviruses, in general, have evolved to interact with the ubiquitinously present HSPGs to gain access to a high affinity receptor. Another focus of our future studies is therefore to study cellular signaling upon Ad-HSPG interaction *in vitro* and *in vivo*.

## Materials and Methods

### Cell lines

293 (Microbix, Toronto, Ontario, Canada), A549, K562 and HeLa (American Type Culture Collection, ATCC) were cultured in Dulbecco modified Eagle medium (DMEM) supplemented with 10% fetal bovine serum (FBS). Y79 and Ramos (ATCC) cells were maintained in RPMI 1640 medium supplemented with 20% FBS, 1 mM sodium pyruvate, and 10 mM HEPES. CHO-K1, CHO-pgsA-745, CHO-pgsE-606 (ATCC) and CHO-C2 cells (provided by John Atkinson, Washington University, St. Louis, MO) were cultured in minimal essential medium (MEM) supplemented with 10% FBS, 200 µM asparagine, and 200 µM proline. All of the media described above were additionally supplemented with 2 mM L-glutamine, 100 U penicillin/ml, and 100 µg streptomycin/ml (Pen-Strep).

### Viruses

Ad3 (GB strain) and Ad35 (Holden strain) were obtained from the ATCC. Ad3 was also generously provided by Dr. Silvio Hemmi (Institute of Molecular Biology, University of Zürich, Switzerland) and found to be identical with the GB strain from the ATCC as determined by sequencing of the viral genomes. Ads were propagated in 293 cells, *methyl*-^3^H thymidine-labeled, purified, dialyzed and stored in aliquots as described elsewhere [40],[41]. Wild-type Ad particle (viral particle, VP) concentrations were determined spectrophotometrically by measuring the optical density at 260 nm (OD_260_) and plaque titering (plaque forming units, pfu) was performed using 293 cells as described elsewhere [40]. The pfu∶VP ratios for Ad3 and Ad35 were both 1∶15. Multiplicities of infection (MOIs) are stated as pfu per cell for CPE and MTT assays and as VP per cell for internalization and attachment assays.

### Antibodies, recombinant fiber knobs, soluble CD46, and wheat germ agglutinin

Monoclonal antibodies (mAbs) directed against CD46 (clone MEM-258; Serotec), CD80 (L307.4; PE-labeled; BD Pharmingen, San Jose, CA), CD86 (clone 2331; PE-labeled; BD Pharmingen), and HSPG (clone F58-10E4; Seikagu) were used for flow cytometry. The knob domains of Ad3 and Ad35 fibers were produced in *E. coli* with N-terminal tags of six consecutive histidine residues (6-HIS), using the pQE30 expression vector (Qiagen, Valencia, CA) and purified by Ni-NTA agarose chromatography as described elsewhere [35]. The fiber knob proteins were dialyzed against 5 mM KCl, 17% glycerol, and 10 mM MgCl_2_. Soluble CD46 was produced in 293 cells stably transfected with soluble CD46 expression plasmid as described elsewhere [35]. FITC-labeled wheat germ agglutinin was purchased from Vector Laboratories (Burlingame, CA).

### Western blot

Recombinant Ad3 and Ad35 knobs (1 µg respectively) were separated by polyacrylamide gel electrophoresis and then transferred onto nitrocellulose membranes. Protein samples were loaded in loading buffer (50 mM Tris-HCl, pH6.8, 100 mM dithiothreitol, 2% sodium dodecyl sulfate, 10% glycerol, 0.2% bromophenol blue) without boiling. To detect whether recombinant Ad3 and Ad35 knobs bind to CD46, the blot was incubated with sCD46 in TBS (10 mM Tris-HCl, pH 7.5, 150 mM NaCl) and 3% blotting grade milk (BIO-RAD, Hercules, CA) for 1 h at room temperature (RT) and then washed three times for 10 min in TBS-0.05% Tween20 (TBS-T) buffer. The blot was then incubated with anti-CD46 antibody (clone J4.48; Fitzgerald, Concord, MA) (1∶50) in TBS and 3% milk for 1 h at RT and then washed three times for 10 min in TBS-T buffer. To visualize binding, the blot was incubated with goat anti-mouse immunoglobulin G (IgG)-horseradish peroxidase (HRP) (BD Pharmingen) (1∶1000) in TBS and 3% blotting grade milk for 1 h at RT. To detect whether recombinant Ad3 and Ad35 knobs bind to Heparin, the blots were incubated with Heparin-biotin (Sigma) (1∶1000) in TBS for 1 h at RT and then washed three times for 10 min in TBS-T. The blot was then incubated with Streptavidin-HRP (eBioscience, San Diego, CA) (1∶250) in TBS and 3% milk for 1 h at RT and then washed three times for 10 min in TBS-T buffer. Finally, blots were subjected to enhanced chemiluminescence substrate (Pierce, Rockford, IL).

### Flow cytometry

Adherent cells were detached by treatment with Versene (Gibco). After being washed, cells were resuspended in 120 µl of wash buffer (WB; phosphate-buffered saline-1% fetal bovine serum) and incubated for 45 min at 4°C with mAbs (final concentration, 1 µg/ml). Subsequently, cells were washed with WB twice. For CD46 and HSPG staining cells were subsequently incubated with Alexa Fluor 488 goat anti-mouse antibody (Molecular Probes, Invitrogen Corporation, Carlsbad, CA) for 30 min at 4°C. After incubation with the secondary antibody, cells were washed two times with WB. Control samples for CD46 and HSPG staining were incubated with the isotype control as a primary antibody (final concentration, 1 µg/ml) and Alexa Fluor 488 goat anti-mouse as a secondary antibody. Control samples for CD80 and CD86 staining were incubated with PE-labeled isotype control (final concentration, 1 µg/ml). Geometric mean fluorescence intensities were determined via flow cytometry using 10^4^ cells per sample and a FACS scan machine (BD).

### Fiber knob and virus attachment assays

All knob and virus attachment assays were carried out in a final volume of 100 µl in ice-cold adhesion buffer (DMEM supplemented with 2 mM MgCl_2_, 1% FBS, and 20 mM HEPES) containing 10^5^ cells.

#### Ad fiber knob attachment assay

Cells were grown to approximately 60–80% confluency, harvested with Versene, washed twice with PBS. Portions of 10^5^ cells were incubated with Ad3 knob and Ad35 knob or without Ad knob in attachment buffer for 1 h at 4°C. 1000 ng knob/10^5^ cells ( = 9.41×10^6^ knob trimers per cell) was used unless indicated otherwise. Cells were then washed twice in ice-cold (4°C) attachment buffer. Cells were next incubated with anti-Penta-His antibody (Qiagen), in washing buffer (WB, PBS-1%FBS) for 1 h at 4°C, washed twice in WB and incubated with AlexaFuor488 goat anti-mouse IgG antibody (Molecular Probes, Eugene, OR), in WB for 1 h at 4°C. Cells were washed twice in cold WB. Control samples were incubated without knob and with anti-Penta-His primary antibody and AlexaFuor488 goat anti-mouse IgG secondary antibody. Background fluorescence of control samples was subtracted from fluorescence of knob-incubated samples. Geometric mean fluorescence intensities were determined via flow cytometry using 10^4^ cells per sample and a FACS Scan machine (BD).

#### Ad virus attachment assay

Cells were detached from culture dishes by incubation with Versene and washed with PBS. A total of 10^5^ cells/tube were resuspended in 100 µl of ice-cold adhesion buffer containing ^3^H-labeled Ad at a multiplicity of infection (MOI) of 8000 VP per cell. After 1 h of incubation at 4°C, cells were pelleted and washed twice with 0.5 ml of ice-cold WB. After the last wash, the supernatant was removed and the cell-associated radioactivity was determined with a scintillation counter. The number of VP bound per cell was calculated by using the virion specific radioactivity and the number of cells. Background scintillation was determined using cells that were not incubated with ^3^H-labeled Ad. Background scintillation was subtracted from scintillation of ^3^H-labeled Ad incubated samples.

#### Competition assays

The following modifications from the attachment protocols described above were included in order to compete Ad and/or Ad fiber knob binding: (i) For knob competition of virus binding, various concentrations (0.01 to 20 µg/ml) of fiber knob were allowed to attach for 45 min at 4°C in attachment buffer. Non-bound knob was removed by washing cells twice with WB before cells were resuspended in attachment buffer containing ^3^H-labeled Ad; (ii) For Heparin competition ^3^H-labeled Ad or fiber knob was incubated in 50 µl attachment buffer supplemented with 50 µl Heparin-Sodium solution (stock concentration 1000USP Units/ml; American Pharmaceutical Partners Inc., Schaumburg, IL) in a total of 100 µl for 1 h at RT and 5 min on ice before adding to cells; (iii) For EDTA competition cells were pretreated with 10 mM final concentration of EDTA for 15 min at RT in attachment buffer and incubated at 5 min on ice before ^3^H-labeled Ad or fiber knob was added; (iv) For Trypsin competition cells were pretreated with Trypsin-0.1%EDTA (Gibco) solution for 15 min at 37°C and washed twice with ice-cold WB before ^3^H-labeled Ad or fiber knob was added; (v) For Heparinase I competition cells were preincubated with 1 U Heparinase I (Sigma, St. Louis, MO) in 100 µl PBS for 1 h at 37°C and 5 min on ice and washed twice with ice-cold PBS before ^3^H-labeled Ad or fiber knob was added; (vi) For Neuraminidase competition cells were pre-incubated with 20 mU Neuraminidase (Sigma) in 100 µl PBS for 1 h at 37°C and 5 min on ice and washed twice with ice-cold WB before ^3^H-labeled Ad or fiber knob was added.

### Virus internalization assay

5×10^4^ CHO cells per well were seeded in 24 well plates and 24 h later ^3^H-labeled Ad was added at multiplicities of infection (MOIs) of 15,360 VP/cell (Ad3) or 153,600 VP/cell (Ad35). Five days post-infection cells were detached and surface bound viral particles were removed using incubation with Trypsin-0.1%EDTA (Gibco) for 10 min. Cells were washed twice with 0.5 ml of ice-cold WB. After the last wash, the supernatant was removed and the cell-associated radioactivity was determined with a scintillation counter. The number of VP internalized per cell was calculated by using the virion specific radioactivity and the number of cells. Background scintillation was determined using cells that were not incubated with ^3^H-labeled Ad. Background scintillation was subtracted from scintillation of ^3^H-labeled Ad incubated samples.

### Virus replication assays

5×10^4^ CHO or A549 cells were seeded in one ml medium per well in 24 well plates and 24 h later infected with multiplicities of infection (MOIs) of 100 pfu/cell (Ad3 and Ad35, A549 cells), 1024 pfu/cell (Ad3, CHO cells), and 10240 pfu/cell (Ad35, CHO cells). Cells (adherent and floating) and supernatants were collected at time points 0 h and 5 days post-infection for quantification of Ad3 and Ad35 genomes and plaque forming units in order to test for viral replication.

#### Viral genomes

Ad3 and Ad35 genomes (genomes per ml) were quantified 0 h and 5 days post-infection. Total DNA from individual samples (supernatant and cells) was extracted using the Blood & Cell Culture DNA Mini Kit (Qiagen). Before DNA extraction each sample was supplemented with 2×10^6^ non-infected CHO-K1 cells to provide carrier DNA. Ad3 and Ad35 viral genomes were subsequently quantified via real-time PCR using a LightCycler (Roche) and a QuantiTect Sybr Green PCR Kit (Qiagen). The following primer pairs located within the fiber encoding regions were used: Ad3-F, 5′-AGCTCGGCTAAGCACTTCCT-3′; Ad3-R, 5′–GGAGCCGCTTGCAGTGGTAA -3′ (168 bp amplicon, PubMed accession# AY599834); Ad35-F, 5′- TCTTCTACAGCGACCAGTGA -3′; Ad35-R, 5′- ATGGCATAGGCAACATTGGA -3′ (211 bp amplicon, PubMed accession# AC_000019). Samples were equalized for DNA input using control primers against the hamster 16S ribosomal RNA gene: HA-16S-F: 5′- CGAAACCAAACGAGCTACCTA-3′; HA-16S-R: 5′-TGGGTAACCAGCTATCACCA -3′ (121 bp amplicon, PubMed accession# AY011148). Dilution series of purified Ad DNA and cellular DNA were used as standard curves. Specificity of amplification products was confirmed using melting curve analysis and agarose gel electrophoresis. PCR conditions were 15 min at 95°C followed by 45 amplification cycles (20 sec at 60°C, 20 sec at 72°C and 15 sec at 95°C).

#### Plaque forming units

Ad3 and Ad35 plaque forming units (pfu per ml) were quantified 0 h and 5 days post-infection. Samples (supernatant and cells) were incubated in liquid nitrogen and at 37°C four consecutive times to release viral particles from cells. Subsequently, pfu titering was performed as described above (see Viruses).

### Immunohistochemistry

1.25×10^4^ CHO-K1 cells were seeded per well in Lab-Tek 8-well chamber glass slides (Nalge Nunc International, Rochester, NY). 24 h later cells were infected with various MOIs of Ad3 (0, 64, 128, 256, 512, 1024 pfu/cell) or Ad35 (0, 640, 1280, 2560, 5120, 10240 pfu/cell). Three days post-infection, cells were fixed with Acetone/Methanol and washed twice with PBS. Slides were blocked for 20 min at RT using PBS-5% blotting grade milk (BIO-RAD, Hercules, CA) followed by incubation with Cy3-labeled mouse anti-hexon antibody (concentration 1∶100; clone 20/11; Chemicon International) and FITC-labeled mouse anti-E-cadherin antibody (concentration 1∶100; clone 36/E-Cadherin, BD Pharmingen) in PBS for 1 h at RT. Slides were washed twice with PBS, mounted with Mounting Medium for Fluorescence (with DAPI; Vector Laboratories) and then analyzed using a fluorescence microscope.

### CPE assay

#### Human cells

1×10^5^ HeLa or A549 cells were seeded per well in 24 well plates and incubated 24 h later with 4 U/ml Heparinase I (Sigma) for 6 h followed by infection with Ad3 and Ad35 using multiplicities of infection (MOIs) ranging from 2–100 pfu/cell.

#### CHO cells

1.25×10^4^ CHO-K1, CHO-pgsA-745 or CHO-pgsE-606 cells were seeded per well in 96 well plates and 24 h later infected with MOIs ranging from 64–2048 pfu/cell (Ad3) or 640–20480 pfu/cell (Ad35).

Cells were continuously monitored for CPE as a sign for viral infection. CPE was defined as the presence of both a discontinuous cellular monolayer (“gaps”) and detached (“round”) cells at the same time. Cells were photographed via microscopy (magnification 40×) after crystal violet staining. For crystal violet staining CHO cells were fixed 5 days after infection with 4% paraformaldehyde for 10 min at room temperature. Fixed cells were incubated for 10 min in 1% crystal violet in 70% ethanol, followed by three rinses with water.

### MTT assay

1.25×10^4^ CHO-K1, CHO-pgsA-745 or CHO-pgsE-606 cells were seeded per well in 100 µl medium in 96 well plates and 24 h later infected with MOIs ranging from 64–1024 pfu/cell (Ad3) or 640–10240 pfu/cell (Ad35). 7 days post-infection 20 µl of MTT stock solution (stock concentration 5 mg/ml in PBS) was added in each well and cells were incubated for 2 h at 37°C. Medium was removed and cells were washed twice with PBS and then air-dried. 100 µl of DMSO/well was added and incubated for 30 min at RT in order to dissolve crystals. Absorbance was measured in plate reader at 546 nm.

### Glycan array

We used a high-throughput glycan array developed by cores D and H of the Consortium for Functional Glycomics (CFG; an NIH National Institute of General Medical Sciences initiative) for identifying specific carbohydrate binding partners for proteins. The glycan binding specificities of Ad3 and Ad35 recombinant fiber knob were screened. The printed array (Version 3.0) contained 320 different natural and synthetic glycans (including sialylated sugars with different linkages and modifications, for example, sulfation, but not heparin sulfate; http://www.functionalglycomics.org/glycomics/publicdata/microarray.jsp). The method used for generating the printed array is detailed in a publication by Blixt et al. [42]. Briefly, the array is created using a robotic printing technology that uses amine coupling to covalently link amine-functionalized glycans or glycanconjugates to amine-reactive *N*-hydroxysuccinimide-activated glass slides. The slides contain six addresses per glycan or glycoconjugate. A printed slide was incubated with Ad3 or Ad35 knob (100 µg/ml), and then an anti-Penta-His monoclonal antibody (Qiagen) (1 µg/ml) was overlaid on the bound knobs followed by a goat anti-mouse AlexaFluor488-labeled secondary antibody (Molecular Probes) (1 µg/ml). The fluorescence intensity was detected using a ScanArray 5000 (Perkin-Elmer Inc.) confocal scanner. The image was analyzed using the IMAGENE image analysis software (BioDiscovery, El Segundo, CA). The data were plotted using the Microsoft EXCEL software.

### Statistical analysis

Statistical significance was calculated by two-sided Student's *t*-test. *P*-values <0.05 were considered statistically significant.

## Supporting Information

Figure S1 Competition of Ad3 and Ad35 virus particle attachment to 293 cells using pre-incubation of cells with increasing concentrations of the corresponding knob proteins. Data points represent the mean and standard deviation of experiments performed in triplicate. This experiment was independently repeated once with a similar outcome.
(1.26 MB TIF)

Figure S2 Detection of Ad3 and Ad35 fiber knob binding to HeLa cells. Overlays of representative flow cytometry charts are shown. 1×10^5^ HeLa cells were incubated with increasing amounts of Ad3 and Ad35 knob (0–2000 ng per 1×10^5^ cells) as indicated by different colors. For mean fluorescence intensities and standard deviations, see Figure 1B.
(1.70 MB TIF)

Figure S3 Competition of Ad3 knob, Ad35 knob, and wheat germ agglutinin attachment to HeLa cells using Neuraminidase pre-incubation of cells. Bars represent the mean and standard deviation of experiments performed in duplicate.
(1.19 MB TIF)

Figure S4 Detection of CD46, HSPG, CD80, and CD86 surface expression on HeLa, A549, K562, and Ramos cells. Overlays of representative flow cytometry charts are shown.
(1.80 MB TIF)

Figure S5 Ad3 and Ad35 internalization assay in CHO-K1, CHO-pgsA-745, and CHO-pgsE-606 cells. ^3^H-labeled Ad3 and Ad35 viral particles internalized per cell 5 days post-infection are shown. Bars represent the mean and standard deviation of experiments performed in duplicate.
(1.27 MB TIF)

Figure S6 Ad3 and Ad35 viral replication assay. Fold increase of plaque-forming units 5 days post-infection of CHO-K1, CHO-pgs-745, CHO-pgsE-606, and A549 cells is shown. Bars represent the mean and standard deviation of experiments performed in duplicate.
(1.33 MB TIF)
