# Supplementary figures and images for: Role of Cellular Heparan Sulfate Proteoglycans in Infection of Human Adenovirus Serotype 3 and 35
Source: PLoS Pathog. 2008 Oct 31;4(10):e1000189. doi: 10.1371/journal.ppat.1000189 (PMC2568953; doi:10.1371/journal.ppat.1000189)

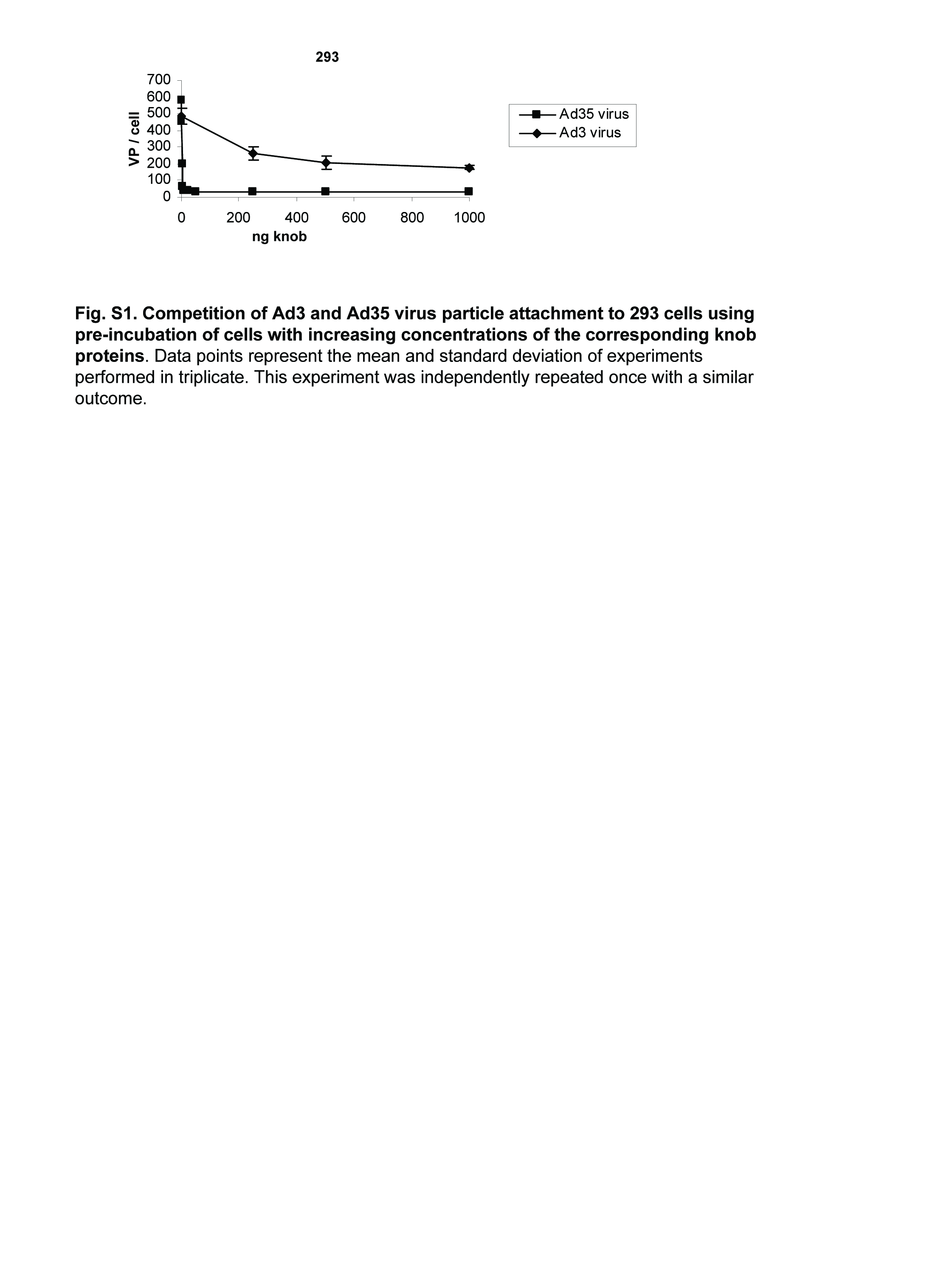

Supplement: Figure S1 — Competition of Ad3 and Ad35 virus particle attachment to 293 cells using pre-incubation of cells with increasing concentrations of the corresponding knob proteins. Data points represent the mean and standard deviation of experiments performed in triplicate. This experiment was independently repeated once with a similar outcome. (1.26 MB TIF) [file ppat.1000189.s001.tif]

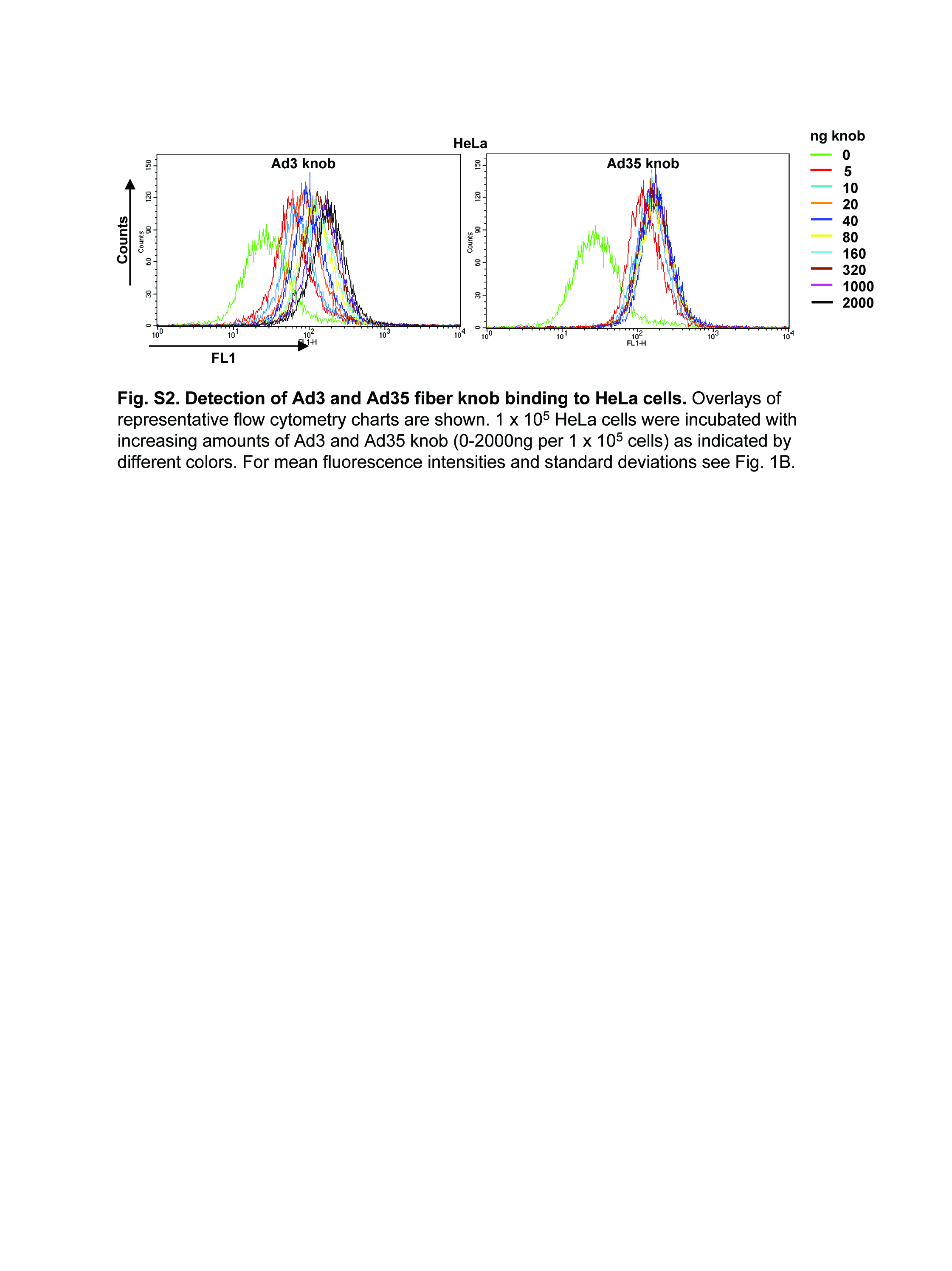

Supplement: Figure S2 — Detection of Ad3 and Ad35 fiber knob binding to HeLa cells. Overlays of representative flow cytometry charts are shown. 1×10^5^ HeLa cells were incubated with increasing amounts of Ad3 and Ad35 knob (0–2000 ng per 1×10^5^ cells) as indicated by different colors. For mean fluorescence intensities and standard deviations, see Figure 1B. (1.70 MB TIF) [file ppat.1000189.s002.tif]

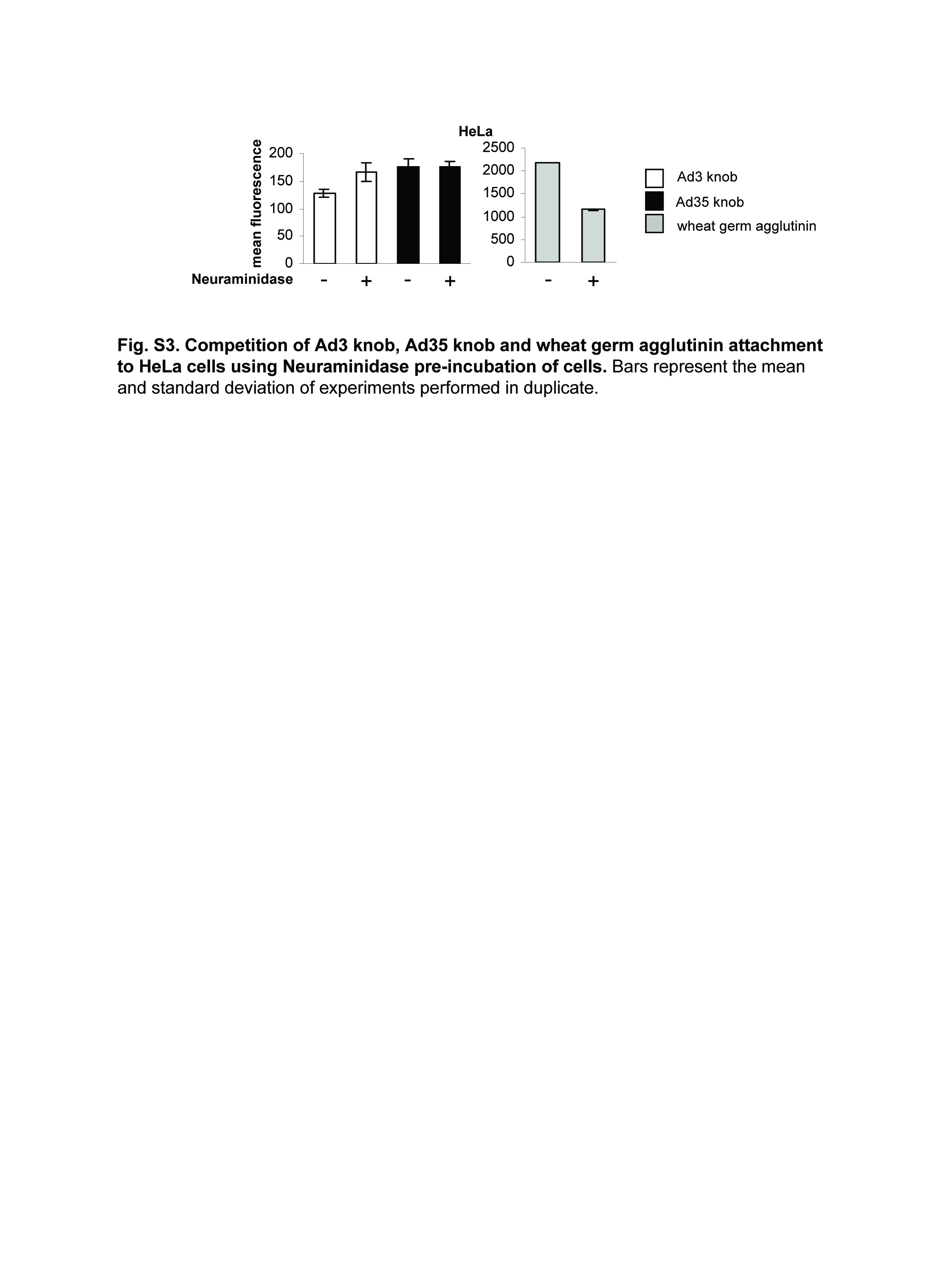

Supplement: Figure S3 — Competition of Ad3 knob, Ad35 knob, and wheat germ agglutinin attachment to HeLa cells using Neuraminidase pre-incubation of cells. Bars represent the mean and standard deviation of experiments performed in duplicate. (1.19 MB TIF) [file ppat.1000189.s003.tif]

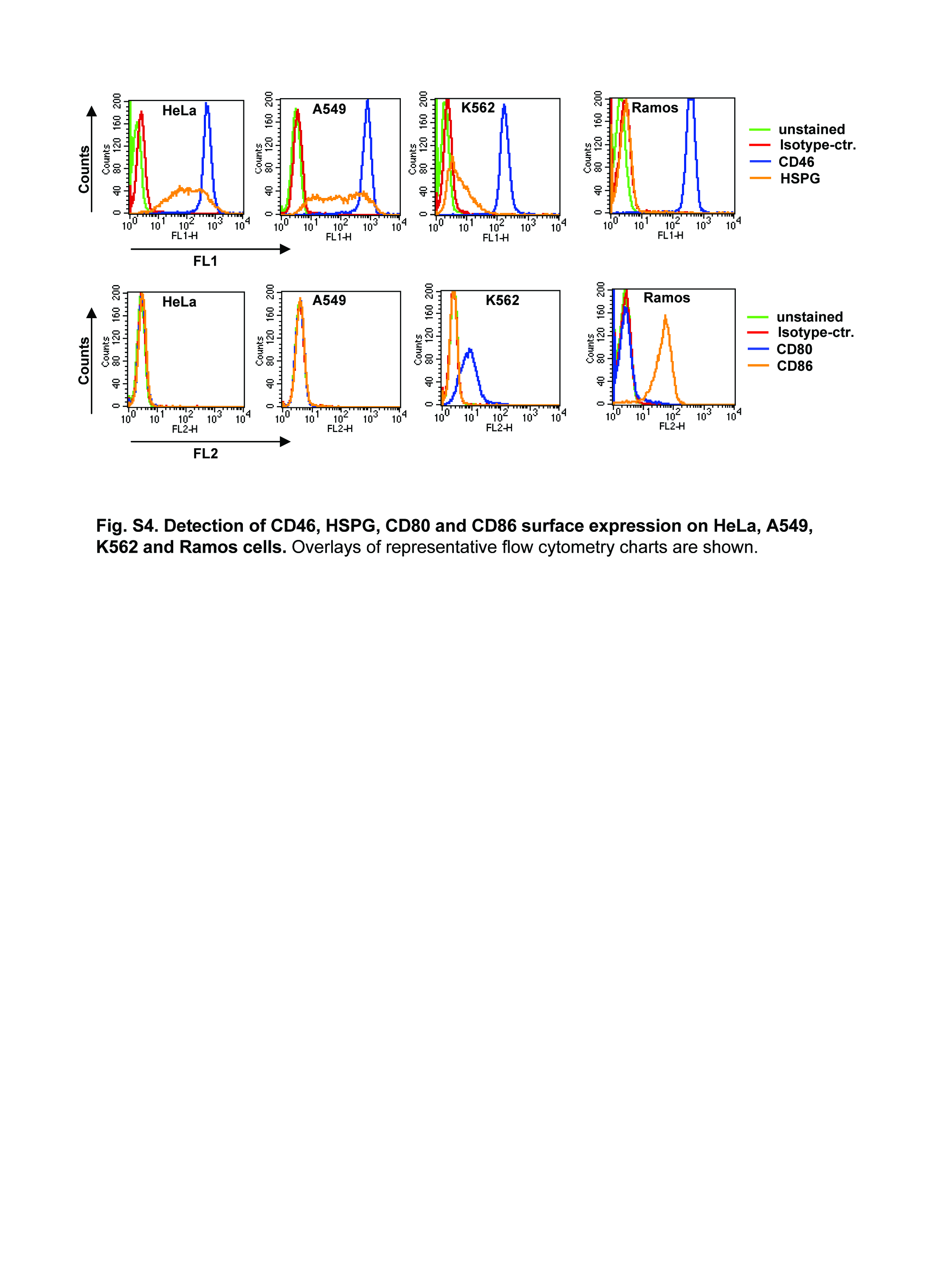

Supplement: Figure S4 — Detection of CD46, HSPG, CD80, and CD86 surface expression on HeLa, A549, K562, and Ramos cells. Overlays of representative flow cytometry charts are shown. (1.80 MB TIF) [file ppat.1000189.s004.tif]

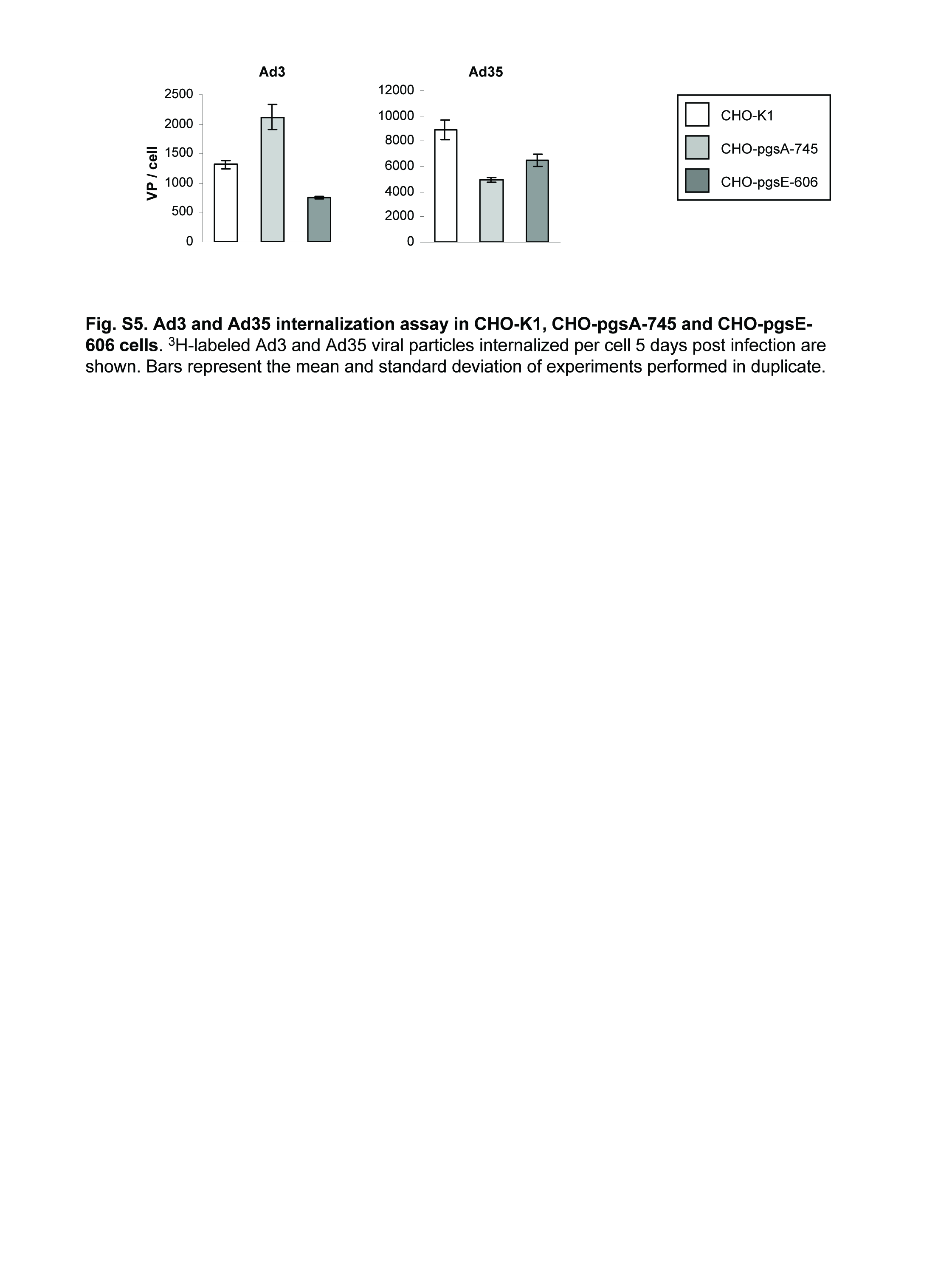

Supplement: Figure S5 — Ad3 and Ad35 internalization assay in CHO-K1, CHO-pgsA-745, and CHO-pgsE-606 cells. 3H-labeled Ad3 and Ad35 viral particles internalized per cell 5 days post-infection are shown. Bars represent the mean and standard deviation of experiments performed in duplicate. (1.27 MB TIF) [file ppat.1000189.s005.tif]

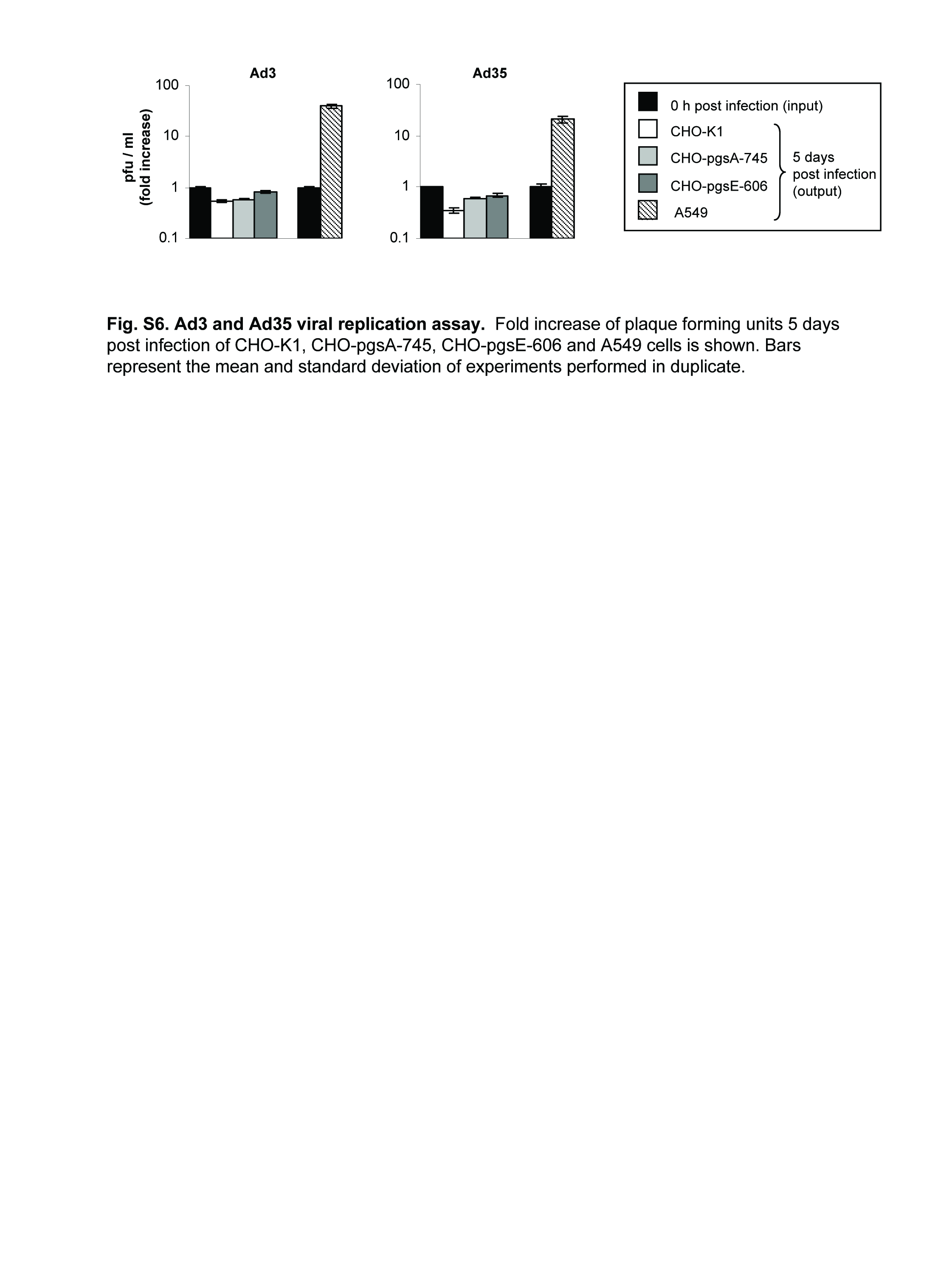

Supplement: Figure S6 — Ad3 and Ad35 viral replication assay. Fold increase of plaque-forming units 5 days post-infection of CHO-K1, CHO-pgs-745, CHO-pgsE-606, and A549 cells is shown. Bars represent the mean and standard deviation of experiments performed in duplicate. (1.33 MB TIF) [file ppat.1000189.s006.tif]
